# Population health interventions for cardiometabolic diseases in primary care: a scoping review and RE-AIM evaluation of current practices

**DOI:** 10.3389/fmed.2023.1275267

**Published:** 2024-01-04

**Authors:** Margot Rakers, Nicoline van Hattem, Sabine Plag, Niels Chavannes, Hendrikus J. A. van Os, Rimke C. Vos

**Affiliations:** ^1^Department of Public Health and Primary Care, Leiden University Medical Centre, Leiden, Netherlands; ^2^Health Campus the Hague, Leiden University Medical Center, The Hague, Netherlands

**Keywords:** population health management, cardiometabolic diseases, RE-AIM framework, population health impact score, modifiable risk factors

## Abstract

**Introduction:**

Cardiometabolic diseases (CMD) are the leading cause of death in high-income countries and are largely attributable to modifiable risk factors. Population health management (PHM) can effectively identify patient subgroups at high risk of CMD and address missed opportunities for preventive disease management. Guided by the Reach, Efficacy, Adoption, Implementation and Maintenance (RE-AIM) framework, this scoping review of PHM interventions targeting patients in primary care at increased risk of CMD aims to describe the reported aspects for successful implementation.

**Methods:**

A comprehensive search was conducted across 14 databases to identify papers published between 2000 and 2023, using Arksey and O’Malley’s framework for conducting scoping reviews. The RE-AIM framework was used to assess the implementation, documentation, and the population health impact score of the PHM interventions.

**Results:**

A total of 26 out of 1,100 studies were included, representing 21 unique PHM interventions. This review found insufficient reporting of most RE-AIM components. The RE-AIM evaluation showed that the included interventions could potentially reach a large audience and achieve their intended goals, but information on adoption and maintenance was often lacking. A population health impact score was calculated for six interventions ranging from 28 to 62%.

**Discussion:**

This review showed the promise of PHM interventions that could reaching a substantial number of participants and reducing CMD risk factors. However, to better assess the generalizability and scalability of these interventions there is a need for an improved assessment of adoption, implementation processes, and sustainability.

## 1 Introduction

Cardiometabolic diseases (CMD), which include cardiovascular disease, diabetes mellitus, and chronic renal failure, are the leading cause of death in high-income countries and are increasing worldwide. If this situation continues unchecked it could potentially compromise the sustainability of healthcare systems (1–3). Cardiometabolic diseases can be prevented for a large part by addressing modifiable risk factors, such as elevated blood pressure, unhealthy dietary habits, and smoking (4–7). To accomplish this, the proactive identification of high-risk patients is essential for early detection of these modifiable risk factors (8).

Population health management (PHM) is a strategy that supports proactive care by identifying and addressing missed opportunities in chronic disease management (9). Population Health Management, in a clinical context, is also known as panel management and can be defined as ‘the proactive management of a total population at risk for adverse outcomes through various individual, organizational and cultural interventions based on a risk-stratified needs assessment of the population’ (9). Primary care occupies a central position in the implementation of PHM thanks to its inherent capacity for care coordination and integration, coupled with access to comprehensively coded routine care data. These unique characteristics of primary care also promote the effective identification of individuals at increased risk of CMD progression and the provision of appropriate care related to identified risk (10, 11).

While there is increasing interest in PHM in relation to CMD, a clear overview detailing how PHM interventions are best implemented in primary care is lacking. Although various implementation theories and frameworks are available, the RE-AIM framework provides a vital tool for evaluating and comprehending the effectiveness and sustainability of PHM interventions in primary care. The RE-AIM framework assesses the impact of population health intervention initiatives using five critical factors: Reach, Efficacy, Adoption, Implementation, and Maintenance (12). Additionally, this framework aids in determining the potential population health impact of these interventions.

This scoping review aimed to identify PHM interventions, which were targeted at patients with a high risk of CMD in the primary care setting. This was accomplished by obtaining information according to the dimensions outlined in the RE-AIM framework and estimating their potential population health impact. In doing so, the study has the objective of contributing to an understanding of the implementation of PHM interventions, their potential population health impact, and to better inform future research efforts.

## 2 Methods

### 2.1 Search strategy

This scoping review followed the recommendations of Preferred Reporting Items for Systematic Reviews and Meta-Analyses for Scoping Reviews (PRISMA-ScR) (13). The search was conducted in the electronic databases Pubmed, Embase, Web of Science, COCHRANE Library, Emcare, Academic Search Premier, IEEE Xplore, ACM Digital Library, MathSciNet, AAAi.org, arXiv, Epistemonikos, PsycINFO and Google Scholar. The search was formulated as a combination of terms that included PHM, primary care, and implementation. The terms were identified through searches of the National Library of Medicine MeSH Tree Structures and by the review team expert. The full search strategy can be found in Supplementary File 1.

### 2.2 Eligibility criteria

Peer-reviewed journal papers were included when they met the following eligibility criteria: (i) published between 2000–2023, (ii) written in English, (iii) published as original results, (iv) focused on risk-based identification of patient groups (panels) with a high risk of (progression of) CMD using a primary care data source or within a primary care setting, and (v) focused on original data about implementation as part of (pragmatic) randomized controlled trials, clinical trials, (retrospective) cohort, case–control, implementation studies, cost-effectiveness or (pilot) feasibility studies. Studies that focused on developing theoretical PHM interventions and those in which the strategy was implemented in a setting other than primary care were excluded.

### 2.3 Data extraction

Using the inclusion and exclusion criteria, two reviewers (M.M.R and S.P.C.P) independently screened all articles on title and abstract. Reading the full text, two team members (M.M.R and S.P.C.P) assessed the selected articles for eligibility. Disagreements were discussed by the core team (M.M.R, N.E.v.H, and S.P.C.P) until consensus was reached. Subsequently, the core team members (M.M.R., N.E.v.H, and S.P.C.P) independently completed full data extraction of study characteristics (publication year, purpose, target population, study design and steps within PHM), and the five dimensions of the RE-AIM framework (14). The three assessors addressed their differences until they came to a complete understanding.

For data extraction focused on RE-AIM dimensions, researchers employed a modified extraction technique created specifically designed for systematic reviews using a RE-AIM framework (see Supplementary File 2) (15, 16). Each of the five RE-AIM dimensions was broken down into a number of components, and the core team (M.M.R., N.E.v.H, and S.P.C.P) categorized each recorded article in relation to whether they reported on specific components. Components were based on inclusion criteria: process interventions to improve clinical health outcomes of a defined group of individuals through proactive care coordination and patient engagement. The components for *Reach* were: the description of the target population, method of identifying the population, recruitment strategies, inclusion/exclusion criteria, participation rate, and cost of the recruitment. For *Effectiveness*, quality of life measures, positive outcomes, unintended or negative consequences, and cost-effectiveness were reported. *Adoption* was extracted based on the site and staff participation rate, description of the intervention location, method of identifying setting and staff, level of expertise of providers, and inclusion or exclusion criteria for providers. *Implementation* was coded on intervention description, theory-based interventions, engagement, consistency of implementation, financial investment, and the number and timing of intervention contacts during implementation. Lastly, *Maintenance* was extracted based on follow-up time, program sustainability, and modifications to the original program. Subsequently, the RE-AIM average [((Participation rate + ESkey outcomes + adoption rate + implementation rate)/4) × 100] was computed by aggregating the scores across the RE-AIM dimensions. This RE-AIM average represented the potential population health impact of the interventions (12, 17).

## 3 Results

### 3.1 Intervention characteristics of the studies reviewed

**Figure 1:**
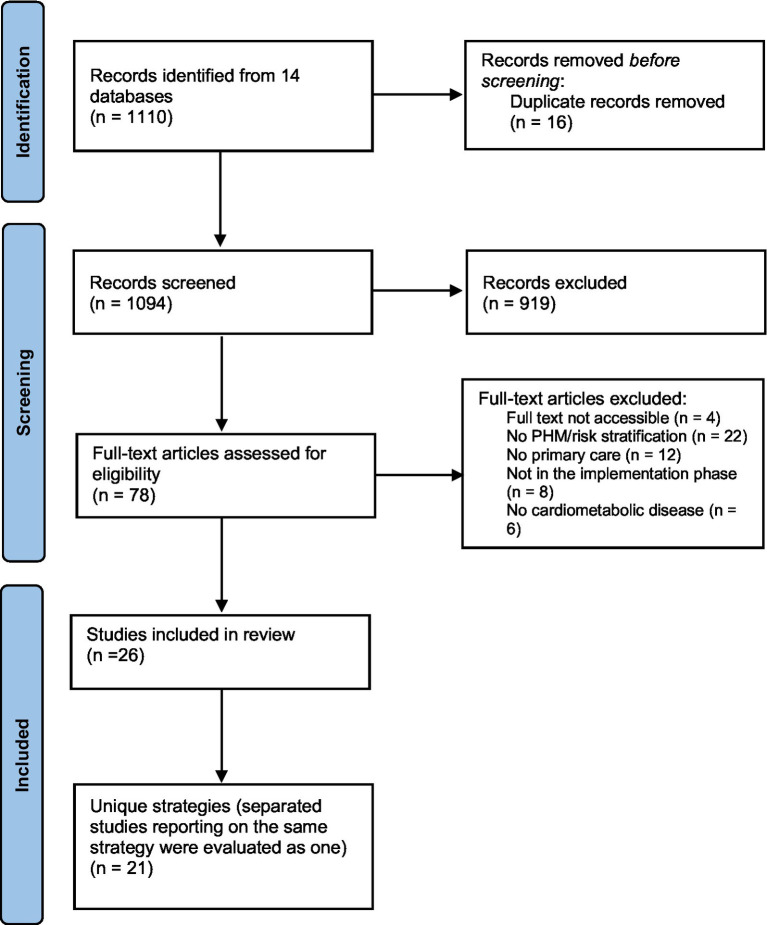
Preferred reporting items for systematic reviews and meta-analyses flow chart, which included searches of databases.

Of the 1,110 studies initially identified, 78 remained after removing duplicates and screening titles and abstracts. Full-text screening led to the inclusion of 26 studies, representing 21 unique interventions (see Figure 1). Most PHM interventions were published in the last five years (13 of 21). Seven of the 21 included interventions were randomized controlled studies, and eight were prospective cohort studies. The characteristics of the reviewed interventions included in the analysis are summarized in Table 1.

**Table 1:** PHM intervention characteristics of studies reviewed ordered chronologically.

Intervention (+ companion publications)^a^	Intervention location	Intervention focus	Target population and Sample size (*n*)	Study design	Primary outcome	Sig. outcomes
Singh et al., 2022 (18)	United Kingdom	West Hampshire Improving Shared Diabetes Outcome Measures (WISDOM) self-management intervention	40.548 DM2patients receiving WISDOM	Difference-in-difference analysis	Diabetes-related complications, quality-adjusted life years (QALYs) and costs	Yes
Ross et al., 2022 (19)	United Kingdom	DDPP (Digital stream of diabetes prevention program)	3,623 non-diabetic hyperglycemia patients	Prospective cohort design	HbA1c and weight changes at 12 months	Yes
Plutzky et al., 2022 (20)	USA	Guideline-directed cholesterol management	1,021 high atherosclerotic CVD risk patients	Prospective cohort design	Program-achieved LDL-C levels	Yes
Sidebottom et al., 2021 (21)^a,1^	USA	Heart of New Ulm (HONU) Project, a rural population-based CVD prevention initiative	CVD risk patients, 4.056 residents of New Ulm matched with 4,056 residents from a different community	Prospective cohort design	Major CVD events	No
Wilson et al., 2021 (22)	USA	PHM approach to recruit participants to a diabetes trial	599 diabetes patients	RCT	Reach and representativeness	NM
Hickey et al., 2021 (23)	Kenya & Uganda	Evaluate effect of patient-centered, streamlined care intervention	32 communities, 10.928 patients with uncontrolled hypertension	RCT	3-year all-cause mortality	Yes
Kozlowska et al., 2020 (24)	United Kingdom	Collaborative diabetes care between primary, secondary and community care	Eighteen virtual clinics across seven teams, 150 patients with diabetes at risk of developing complications	Pilot feasibility study	Acceptability, feasibility and short-term impact	NM
Baer et al., 2020 (25)	USA	Combined intervention, including an online weight management program plus PHM.	840 patients with BMI between 27–40 and hypertension or type 2 diabetes	RCT	Weight change at 12 months	Yes
Cykert et al., 2020 (26)	USA	PHM intervention with practice facilitation and risk-stratification	146,826 high risk CVD patients	RCT	Change in the average 10-year CVD risk score	Yes
Jazowski et al., 2020 (27)^a,2^	USA	Team-supported, Electronic health record (EHR)–leveraged, Active Management (TEAM)	62 patients with uncontrolled hypertension	Pilot study	Feasibility changes in blood pressure	NM
Jølle et al., 2018 (28)	Norway	Basic lifestyle advice	2,380 high risk DM2 patients	Prospective cohort design	2-year diabetes risk	No
Van Houtven et al., 2018 (29)	USA	Southeastern Diabetes Initiative (SEDI)	65,683 patients with prevalent DM2	Pre-post cohort design	Utilization, screening, and costs	Yes
Wan et al., 2018 (30)^a,3^	Japan	Risk Assessment and Management Program–Diabetes Mellitus (RAMP-DM)	RAMP-DM group 29,396 patients; usual care group 29,396 DM2 patients	Prospective cohort design	All-cause mortality	Yes
Ashburner et al., 2017 (31)	USA	Health information technology-enabled PHM program for chronic disease management	66,091 patients with diabetes, CVD or hypertension	Prospective cohort design	Changes in diabetes, CVD, hypertension measures at 6 months	Yes
Price-Haywood et al., 2017 (32)	USA	Collaborative care models incorporating pharmacists	5,044 patients with diabetes and/or hypertension with high risk for disease complications	Retrospective cohort design	A1c level for diabetics and BP for patients with hypertension	No
Emerson et al., 2016 (33)	USA	PHM incorporating telemedicine tools and health coaches	Ten poorly-controlled diabetic patients	Pilot RCT	Feasibility of protocol implementation	NM
Yu et al., 2016 (34)	Japan	Multidisciplinary risk assessment and management program for patients with hypertension (RAMP-HT)	20,524 patients with hypertension	Longitudinal cohort study	Proportion of patients achieving satisfactory blood pressure	Yes
Schwartz et al., 2015 (35)^a,4^	USA	Incorporation of PMA into primary care teams	8,150 patients with hypertension and/or smoking	RCT	Hypertension and smoking variables	No
Krantz et al., 2013 (36)^a,4^	USA	Prevention CVD program with community health workers	698 CVD risk patients	Prospective cohort design	Change baseline 10-year FRS	Yes
Evans et al., 2010 (37)	Canada	Collaborative pharmacist intervention that used a systematic case-finding procedure	176 high risk CVD patients	Pilot RCT	Mean reduction in the 10-year Framingham risk score	No
Clark et al., 2001 (38)	USA	Diabetes management program that included risk stratification and social marketing	370 patients with diabetes	Prospective cohort design	Clinical diabetes outcomes	Yes

RCT, randomized controlled trial; Sig, statistically significant; NM, not measured; CVD, cardiovascular disease; LDL-C, low-density-lipoprotein cholesterol; BMI, body mass index; DM, diabetes mellitus; PMA, Panel Management Assistants; FRS, Framingham Risk Score. ^a^Interventions with separate studies in which additional information was found: ^1^Sidebottom et al., 2016 (39), Sidebottom et al., 2021 (21); ^2^Lewinski et al., 2021 (40); ^3^Jiao et al., 2014 (41); ^4^Strauss et al., 2015 (42); ^5^Smith et al., 2019 (43).

### 3.2 RE-AIM evaluation

A comprehensive overview of RE-AIM dimensions, including individual components, can be found in Table 2 (detailed data extraction is provided in Supplementary File 3). Three of the PHM interventions only reported data on (or at least one of the individual components of) three dimensions: reach, adoption and implementation. Another 12 interventions reported data on four dimensions: reach, effectiveness, adoption and implementation. Six interventions provided information on all five dimensions of the RE-AIM framework.

**Table 2:** Number of interventions reporting the RE-AIM dimensions.

RE-AIM dimensions (and components)	Number of interventions reporting *n* (%)	Interventions
Reach		
Description of the target population	21 (100)	(18–20, 22–29, 31–38, 44)
Method to identify the target population	20 (95)	(19, 20, 23–38, 44, 45)
Recruitment strategies	20 (95)	(18–20, 22–30, 32–38, 44)
Inclusion/exclusion criteria for individuals	20 (95)	(19, 20, 22–29, 31–38, 44)
Individual participation rate	19 (91)	(19, 20, 22–31, 33, 34, 36–38, 44)
Cost of recruitment	3 (14)	(22, 23, 28)
Qualitative methods to measure reach	1 (5)	(24)
Effectiveness		
Positive outcomes	19 (91)	(18–20, 23–27, 29–38, 44)
Quality of life	1 (5)	(18)
Negative consequences	2 (10)	(20, 23)
Cost-effectiveness	6 (29)	(18, 23, 26, 29, 33)
Qualitative methods to measure effectiveness	1 (5)	(24)
Adoption		
Site participation rate	17 (81)	(19, 20, 22–26, 29–33, 35–38, 44)
Description of intervention location	16 (76)	(20, 22, 24–26, 28–33, 35–37, 44, 46)
Method of identifying setting	7 (33)	(18, 20, 22, 23, 25, 26, 34)
Average number of persons served per setting	16 (76)	(19, 20, 22, 25, 26, 29, 31, 33, 35–38, 44)
Staff participation rate	5 (24)	(22, 25, 35, 36, 38)
Method of identifying target providers/staff	2 (10)	(20, 31)
Level of expertise of providers	18 (86)	(18–20, 23–25, 28–38, 44)
Inclusion/exclusion criteria for providers	3 (14)	(19, 20, 35)
Qualitative methods to measure adoption	1 (5)	(24)
Implementation		
Intervention description	21 (100)	(18–20, 22–38, 44)
Theory-based	2 (10)	(27, 36)
Engagement	5 (24)	(19, 25, 26, 36, 44)
Intervention contacts	15 (71)	(19, 20, 23–25, 27–30, 33–38, 44)
Timing of intervention contacts	17 (81)	(18, 19, 22–29, 32–38, 44)
Duration of intervention contacts	8 (38)	(19, 20, 22, 23, 28, 32, 36, 38, 44)
Consistency of implementation across settings or providers	12 (57)	(19, 20, 23–26, 30, 31, 36, 38)
Intervention costs	4 (19)	(18, 23, 29, 33)
Qualitative methods to measure implementation	3 (14)	(24, 25, 33)
Maintenance		
Follow-up outcomes measures at some duration after intervention	0 (0)	
Attrition of individuals	0 (0)	
Maintenance of the program after completion of the study	4 (19)	(27, 29, 33, 38)
Modifications made to the original program	3 (14)	(24, 25, 27)
Attrition of settings	0 (0)	

#### 3.2.1 Reach

Among all the evaluated dimensions in the included interventions, reach was documented most extensively. In total, 15 interventions reported five out of seven reach components (19, 20, 22–25, 27–29, 34, 36–38, 44). All interventions provided information on the target population, and 20 described the method of identifying the target population. The sample size of the interventions ranged from 10 to 146,826 participants, with participation rates varying from 3 to 95%. Five interventions reported a participation rate below 10% (22, 24, 25, 33, 36); the participation rate of 3% was primarily due to non-compliance with inclusion criteria (25). In Mori’s study, the algorithm could not calculate risk for most patients, rendering them unidentifiable. The primary method of participant recruitment for most interventions (11 out of 21) was electronic health record data assessment using algorithms (20, 22, 23, 25, 26, 31, 33, 34, 36, 38, 44), the first step in the panel management approach. However, only a small proportion of interventions (3 out of 21) reported the cost of recruitment activities (22, 23, 28).

#### 3.2.2 Effectiveness

Among the 21 interventions reviewed, nine interventions (43%) focused on patients at risk of (progression of) cardiovascular diseases (20, 23, 26, 27, 34–37, 44), six interventions (29%) on diabetes (18, 19, 22, 24, 28, 33), and six interventions (29%) on CMD (25, 30–33, 38). Of the 12 interventions that evaluated diabetes outcome measurements, only one (5%) reported no statistically significant impact (28), while six reported significantly positive outcomes on diabetes control (18, 19, 25, 30, 31, 38). Two interventions (10%) focused on feasibility and acceptability outcomes and found positive results in terms of better understanding and proficiency in managing individuals with complex diabetes in a primary care setting through PHM (24, 33). Only two interventions reported unintended consequences of the intervention (20, 23), and only one measured quality of life (18). A minority of interventions (24%) addressed the cost of the intervention (18, 23, 26, 29, 33), of which one included a formal cost-effectiveness analysis (18).

#### 3.2.3 Adoption

All included interventions (*n* = 21) documented adoption, but none reported all eight adoption components. The proficiency level of staff was reported in 18 interventions (86%) (18–20, 23–25, 28–30, 32, 34–38, 44), and 19 interventions (91%) described the intervention location (1, 18–20, 24–32, 34–37, 44, 47). However, the staff participation rate and the method of staff identification were mentioned in only five (24%) and two (10%) interventions, respectively. Inclusion or exclusion criteria for staff were documented in three (14%) interventions (19, 20, 35). At the level of the clinical setting, 17 interventions (81%) reported the site participation rate, with an average of 50 to 12,000 individuals served per setting (19, 20, 22–26, 29–31, 33, 35–38, 44, 48). Only one study used qualitative methods to measure adoption, using surveys and observations (24).

#### 3.2.4 Implementation

Descriptions of the intervention were provided for all 21 interventions. Only three interventions (14%) explained the theories or principles that guided the creation of the intervention (27, 34, 36). The frequency, duration, and timing of visits varied across interventions and were sometimes inadequately described. Patient engagement in intervention design was reported in only a single study (25), while five interventions (24%) involved healthcare professionals, experts, and local stakeholders in developing specific intervention components (19, 25, 26, 36, 44).

#### 3.2.5 Maintenance

Maintenance was least often reported across all interventions. Five interventions (24%) reported on the continuation of the program after the study period (27, 29, 30, 33, 41), with just one providing details (27). Additionally, while three interventions (14%) reported modifications to the original program, these changes were implemented during the study period, not after completion (24, 25, 27).

#### 3.2.6 Potential population health impact

Calculating the potential population health impact was possible for six out of 21 interventions (see Table 3), with scores ranging from 45 to 89%. The RE-AIM mean exhibited clear variation attributable to differences within each dimension, except for the implementation score, which remained consistent across all interventions.

**Table 3:** Potential population health impact (RE-AIM average).

Description	Reach (number of participants/number of eligible and invited people)	Efficacy (effect size of the intervention)	Adoption (number of delivery setting/number of eligible and invited settings)	Implementation (consistency of delivering intervention components)	RE-AIM average [(participation rate + ESkey outcomes + adoption rate + implementation rate)/4] × 100
Hickey et al. (23)	10,928/86,078 = 13%	0.21	32/32 = 100%	The 32 practices implement all of the intervention activities.	59%
Ross et al. (19)	3,623/5,053 = 64%	0.5 (weight) 0.8 (HbA1c)	9/8 = 112.5%	The 9 demonstrator sites implement all of the intervention activities.	82% resp. 89%
Plutzky et al. (20)	1,021/1,631 = 63%	0.45	19/19 = 100%	All of the intervention activities are implemented by the 19 practices.	77%
Baer et al. (25)	840/26,393 = 3%	0.29	24/24 = 100%	The 15 practices implement all of the intervention activities.	58%
Cykert et al. (26)	146,826/437,556 = 34%	0.5	219 small primary care practices/801 = 27.3%	All of the intervention activities are implemented by the 219 practices.	56%
Mori et al. (36)	698/4,743 = 15%	0.22	22/22 = 100%	The 20 centers implement all of the intervention activities.	48%

## 4 Discussion

A total of 21 PHM interventions for patients at high risk of CMD in a primary care setting were identified. These interventions showed promise in engaging a substantial number of participants and reducing CMD risk factors. However, this study also revealed a widespread deficiency in reporting across most RE-AIM components. While the included interventions exhibited higher reporting accuracy concerning *Reach*, followed by *Adoption* and *Implementation*, the constructs *Effectiveness* and *Maintenance* were barely addressed. A similar trend was found regarding the population health impact score, as the score could only be calculated for six interventions.

Compared with previous systematic reviews (15, 49–52), *Reach* (especially the description and the method of identifying the target population) was well described, with most interventions using algorithms or risk stratification tools in electronic health records to identify potential participants. Population surveys or routine care checks were employed for those who did not use electronic health records. However, it is worth noting that the risk calculation primarily relied on clinical health outcomes and did not incorporate health behaviors or social determinants of health. Given their significance in determining the risk of a particular group (53, 54), integrating health behaviors and social determinants into the risk model may be crucial to ensure that all potentially suitable patients are included.

Secondly, most studies reported positive outcomes while neglecting to address negative consequences of the intervention adequately. Awareness of negative outcomes, such as attrition and adverse outcomes, is essential for developing effective implementation strategies and ensuring the sustainability of interventions (55). Moreover, most interventions lacked follow-up data and information on attrition, which raises concerns about their long-term effectiveness. However, the short observation period of many interventions may be attributed to the research funding structure, often relying on one-off grants with limited duration and insufficient structural support (56). Nonetheless, long-term results on maintenance and sustainability are crucial for reliable cost-effectiveness analysis, which policymakers and healthcare providers weigh when deciding whether or not to scale up and implement health interventions (16, 57).

Another issue was the lack of comprehensive information regarding *Adoption,* a multifaceted process involving two levels: setting and staff. Specifically, the descriptions of staff involvement were inadequate, potentially leading to a lack of clarity regarding the qualifications necessary to properly implement an intervention. Effective staff involvement is of paramount importance. Previous studies have highlighted the significance of implementation strategies such as education, training, and staff participation in decision-making in promoting successful implementation. Additionally, utilizing champions and opinion leaders to facilitate intervention implementation has been recommended in previous research (16, 58, 59). A lack of reporting on the components of *Adoption* and *Maintenance* makes it challenging to determine whether success can be attributed to the intervention itself, the elements of its implementation, or a combination of both. This consequently limits the prospects of disseminating results and thus extends the reach of an intervention (60).

Finally, an assessment of potential population health impact was conducted for six interventions, utilizing the RE-AIM average score as defined by Glasgow et al. (12). It is important to note that this score does not encompass all facets of the RE-AIM dimensions, necessitating caution in its interpretation. Two interventions resulted in the highest potential population impact scores, which may be linked to their higher participation rates in the *Reach* dimension (19, 20). This can in turn, be attributed to contacting eligible patients via email and telephone, as well as maintenance of extensive intervention contacts. These contacts, including navigator support, website and telephone services, were associated with significant reductions in risk factors for CMD. Moreover, these interventions consistently delivered all components as intended in their respective settings (19, 20).

### 4.1 Limitations of this review

Several limitations need to be taken into account when interpreting the results. Firstly, the search strategy was designed to capture information from English language publications only. Consequently, valuable publications utilizing other languages, housed in other databases, or employing alternative applicable MeSH terms may have been overlooked. Two widely used terms, “panel management” and “PHM,” were utilized to describe the proactive management of an entire population at risk of adverse outcomes. These terms are frequently used interchangeably in the literature, but their recent emergence suggests that a broader search might have yielded more publications. Secondly, current study focused exclusively on the RE-AIM framework and did not explore other frameworks such as the widely used Consolidated Framework for Implementation Research. This decision may have limited the exploration of potential barriers and facilitators to successful implementation. Nonetheless, as the objective was to better understand the potential of interventions for broader dissemination and adoption, the RE-AIM framework was intentionally selected because of its specific emphasis on assessing how interventions perform in real-world implementation settings. We acknowledge that the RE-AIM framework is not the only framework. Rather, it was used as an appropriate framework in which to present carefully systematized findings to enable readers to exercise their own discernment. Finally, another noteworthy limitation pertains to the scope of this review, which was centered on primary care. As the organization of primary care can vary considerably across different countries, it is prudent to exercise caution when applying the findings to countries with different healthcare systems. Nonetheless, it is worth emphasizing that the shared goal of providing accessible and appropriate care to all patients remains a common thread across these diverse settings.

### 4.2 Implications for research and practice

In line with the findings in this study, other health interventions tend to underreport aspects covered by RE-AIM dimensions, which may result in a poor understanding of the factors contributing to the success or failure of intervention implementation (15, 49–52). Providing clear, standardized documentation of the effectiveness of implementation would improve understanding of potential public health impacts and better inform future research efforts (16, 61). Moreover, a better understanding would help demonstrate practical impacts and potentially stimulate wider adoption of such interventions.

Decision-makers can use the population health impact score to assess the feasibility of implementing an intervention within their specific setting (55). However, caution is advised when interpreting an average score because it may not encompass all dimensions outlined in the RE-AIM framework. It may be more insightful to compare the scores for each dimension across different PHM interventions (62). This practical method allows for better visual communication with relevant stakeholders (63), providing a comprehensive view of intervention strengths and weaknesses regarding reach, effectiveness, adoption, implementation, and maintenance.

In conclusion, while many interventions did not fully report results across all RE-AIM dimensions, those that reported on Reach, Effectiveness, Adoption, Implementation, and Maintenance showed positive outcomes. Population Health Management interventions demonstrated their potential by reaching a significant number of participants and reducing CMD risk factors. Assessment of the RE-AIM average indicated that achieving the highest potential population health impact required reaching eligible participants through email or telephone, maintaining extensive intervention contacts via navigator support, website and telephone services, and consistently delivering all intended components within a specific setting. However, to further substantiate these results, reporting on adoption, implementation processes and the sustainability of these interventions must improve.

## Data availability statement

The original contributions presented in the study are included in the article/Supplementary material, further inquiries can be directed to the corresponding author.

## Author contributions

MR: Conceptualization, Formal analysis, Methodology, Project administration, Writing – original draft. NH: Conceptualization, Formal analysis, Writing – review & editing. SP: Formal analysis, Writing – review & editing. NC: Supervision, Writing – review & editing. HO: Funding acquisition, Supervision, Writing – review & editing. RV: Supervision, Writing – review & editing.
