# Supplementary material for: Population health interventions for cardiometabolic diseases in primary care: a scoping review and RE-AIM evaluation of current practices
Source: Front Med (Lausanne). 2024 Jan 4;10:1275267. doi: 10.3389/fmed.2023.1275267 (PMC10794664; doi:10.3389/fmed.2023.1275267)
Supplement: Supplementary file 2 [file Data_Sheet_1.docx]

Supplementary Material

# Supplementary Data

**Search Strategy**

**POPULATION HEALTH MANAGEMENT, CARDIOMETABOLIC DISEASES AND PRIMARY CARE**

May 2023

Update 11-5-2023 - total: 1115 references, including 117 new, from:

- PubMed: 765 - 72 new and unique
- MEDLINE (OVID): 765 - 0 new and unique
- Embase: 808 - 27 new and unique
- Web of Science: 366 - 14 new and unique
- Cochrane Library: 27 - 2 new and unique
- Emcare: 328 - 0 new and unique
- Academic Search Premier: 140 - 2 new and unique

Regular references - total d.d. 13-5-2022: 997 references, from:

- PubMed: 688
- MEDLINE (OVID): 689 - 3 unique
- Embase: 676 - 157 uniqe
- Web of Science: 330 - 113 uniqe
- COCHRANE Library: 23 - 6 uniqe
- Emcare: 349 - 14 uniqe
- Academic Search Premier: 131 - 16 uniqe

Meeting abstract references:

- Embase: 204
- Web of Science: 2
- COCHRANE Library: 12

Databases:

**PubMed**

<http://www.ncbi.nlm.nih.gov/pubmed?otool=leiden>

**Three-pronged strategy –** linked to primary care

- Three components: population health management intervention (main subject), Adult patients at increased risk for cardiometabolic disease, and pilot phase or implemented
- Three components: population health management intervention, Adult patients at increased risk for cardiometabolic disease (main subject), and pilot phase or implemented
- Two components: population health management intervention & Adult patients at increased risk for cardiometabolic disease - emphasis on both components

(**(**((("Cardiovascular Diseases"[mesh] OR "Stroke"[mesh] OR "Myocardial Infarction"[mesh] OR "Brain Infarction"[mesh] OR "Diabetes Mellitus"[Mesh] OR "cardiometabolic disease"[tw] OR "cardiometabolic diseases"[tw] OR "cardio-metabolic disease"[tw] OR "cardio-metabolic diseases"[tw] OR "cardiovascular disease"[tw] OR "cardiovascular diseases"[tw] OR "heart disease"[tw] OR "heart diseases"[tw] OR "stroke"[tw] OR "post-stroke"[tw] OR "TIA"[tw] OR "TIAs"[tw] OR "cerebrovascular accident"[tw] OR "cerebrovascular accident"[tw] OR "CBA"[tw] OR "CVAs"[tw] OR "myocardial infarction"[tw] OR "myocardial infarctions"[tw] OR "myocardial infarct*"[tw] OR "post-myocardial infarction"[tw] OR "brain infarction"[tw] OR "diabetes mellitus"[tw] OR "Diabetes"[tw] OR "IDDM"[tw] OR "NIDDM"[tw] OR "hypertension"[tw]) NOT (("Infant"[mesh] OR "Child"[mesh] OR "Adolescent"[mesh] OR "Infant"[ti] OR "Child"[ti] OR "Adolescent"[ti] OR "Infants"[ti] OR "Children"[ti] OR "Adolescents"[ti]) NOT ("Adult"[mesh] OR "adult"[tw] OR "adults"[ti] OR "elderly"[ti])) AND ("Risk"[mesh] OR "risk"[tw] OR "risks"[tw] OR "atrisk"[tw] OR "outcome"[tw] OR "outcomes"[tw])) AND ("risk stratification tools"[ti] OR "risk stratification tool"[ti] OR "population stratification tool"[ti] OR "population stratification tools"[ti] OR "population segmentation tools"[ti] OR "population segmentation tool"[ti] OR "population health management intervention"[ti] OR "population health management interventions"[ti] OR "empanelment intervention"[ti] OR "panel management intervention"[ti] OR "empanelment interventions"[ti] OR "panel management interventions"[ti] OR "risk stratification"[ti] OR "risk stratif*"[ti] OR "population stratification"[ti] OR "population stratif*"[ti] OR "population segmentation"[ti] OR "population segment*"[ti] OR "Population Health Management"[majr] OR "population health management"[ti] OR "population health manag*"[ti] OR "empanelment"[ti] OR "empanelment*"[ti] OR "panel management"[ti] OR "panel management*"[ti] OR "population-based approach"[ti] OR "population-based"[ti] OR "population panel"[ti] OR "population care"[ti]) AND ("Pilot Projects"[Mesh] OR "Pilot Projects"[tw] OR "Pilot Project"[tw] OR "pilot"[tw] OR "implemented"[tw] OR "implementation"[tw] OR "implement*"[tw] OR "practice use"[tw] OR "practice uses"[tw] OR "clinical practice"[tw] OR "MDR application"[tw] OR "MDR applications"[tw])) **OR** ((("Cardiovascular Diseases"[majr] OR "Stroke"[majr] OR "Myocardial Infarction"[majr] OR "Brain Infarction"[majr] OR "Diabetes Mellitus"[majr] OR "cardiometabolic disease"[ti] OR "cardiometabolic diseases"[ti] OR "cardio-metabolic disease"[ti] OR "cardio-metabolic diseases"[ti] OR "cardiovascular disease"[ti] OR "cardiovascular diseases"[ti] OR "heart disease"[ti] OR "heart diseases"[ti] OR "stroke"[ti] OR "post-stroke"[ti] OR "TIA"[ti] OR "TIAs"[ti] OR "cerebrovascular accident"[ti] OR "cerebrovascular accident"[ti] OR "CBA"[ti] OR "CVAs"[ti] OR "myocardial infarction"[ti] OR "myocardial infarctions"[ti] OR "myocardial infarct*"[ti] OR "post-myocardial infarction"[ti] OR "brain infarction"[ti] OR "diabetes mellitus"[ti] OR "Diabetes"[ti] OR "IDDM"[ti] OR "NIDDM"[ti] OR "hypertension"[ti]) NOT (("Infant"[mesh] OR "Child"[mesh] OR "Adolescent"[mesh] OR "Infant"[ti] OR "Child"[ti] OR "Adolescent"[ti] OR "Infants"[ti] OR "Children"[ti] OR "Adolescents"[ti]) NOT ("Adult"[mesh] OR "adult"[tw] OR "adults"[ti] OR "elderly"[ti])) AND ("Risk"[mesh] OR "risk"[tw] OR "risks"[tw] OR "atrisk"[tw] OR "outcome"[tw] OR "outcomes"[tw])) AND ("risk stratification tools"[tw] OR "risk stratification tool"[tw] OR "population stratification tool"[tw] OR "population stratification tools"[tw] OR "population segmentation tools"[tw] OR "population segmentation tool"[tw] OR "population health management intervention"[tw] OR "population health management interventions"[tw] OR "empanelment intervention"[tw] OR "panel management intervention"[tw] OR "empanelment interventions"[tw] OR "panel management interventions"[tw] OR "risk stratification"[tw] OR "risk stratif*"[tw] OR "population stratification"[tw] OR "population stratif*"[tw] OR "population segmentation"[tw] OR "population segment*"[tw] OR "Population Health Management"[Mesh] OR "population health management"[tw] OR "population health manag*"[tw] OR "empanelment"[tw] OR "empanelment*"[tw] OR "panel management"[tw] OR "panel management*"[tw] OR "population-based approach"[tw] OR "population-based"[tw] OR "population panel"[tw] OR "population care"[tw]) AND ("Pilot Projects"[Mesh] OR "Pilot Projects"[tw] OR "Pilot Project"[tw] OR "pilot"[tw] OR "implemented"[tw] OR "implementation"[tw] OR "implement*"[tw] OR "practice use"[tw] OR "practice uses"[tw] OR "clinical practice"[tw] OR "MDR application"[tw] OR "MDR applications"[tw])) **OR** ((("Cardiovascular Diseases"[majr] OR "Stroke"[majr] OR "Myocardial Infarction"[majr] OR "Brain Infarction"[majr] OR "Diabetes Mellitus"[majr] OR "cardiometabolic disease"[ti] OR "cardiometabolic diseases"[ti] OR "cardio-metabolic disease"[ti] OR "cardio-metabolic diseases"[ti] OR "cardiovascular disease"[ti] OR "cardiovascular diseases"[ti] OR "heart disease"[ti] OR "heart diseases"[ti] OR "stroke"[ti] OR "post-stroke"[ti] OR "TIA"[ti] OR "TIAs"[ti] OR "cerebrovascular accident"[ti] OR "cerebrovascular accident"[ti] OR "CBA"[ti] OR "CVAs"[ti] OR "myocardial infarction"[ti] OR "myocardial infarctions"[ti] OR "myocardial infarct*"[ti] OR "post-myocardial infarction"[ti] OR "brain infarction"[ti] OR "diabetes mellitus"[ti] OR "Diabetes"[ti] OR "IDDM"[ti] OR "NIDDM"[ti] OR "hypertension"[ti]) NOT (("Infant"[mesh] OR "Child"[mesh] OR "Adolescent"[mesh] OR "Infant"[ti] OR "Child"[ti] OR "Adolescent"[ti] OR "Infants"[ti] OR "Children"[ti] OR "Adolescents"[ti]) NOT ("Adult"[mesh] OR "adult"[ti] OR "adults"[ti] OR "elderly"[ti])) AND ("Risk"[mesh] OR "risk"[tw] OR "risks"[tw] OR "atrisk"[tw] OR "outcome"[tw] OR "outcomes"[tw])) AND ("risk stratification tools"[ti] OR "risk stratification tool"[ti] OR "population stratification tool"[ti] OR "population stratification tools"[ti] OR "population segmentation tools"[ti] OR "population segmentation tool"[ti] OR "population health management intervention"[ti] OR "population health management interventions"[ti] OR "empanelment intervention"[ti] OR "panel management intervention"[ti] OR "empanelment interventions"[ti] OR "panel management interventions"[ti] OR (("risk stratification tools"[tw] OR "risk stratification tool"[tw] OR "population stratification tool"[tw] OR "population stratification tools"[tw] OR "population segmentation tools"[tw] OR "population segmentation tool"[tw] OR "population health management intervention"[tw] OR "population health management interventions"[tw] OR "empanelment intervention"[tw] OR "panel management intervention"[tw] OR "empanelment interventions"[tw] OR "panel management interventions"[tw]) AND ("risk stratification"[ti] OR "risk stratif*"[ti] OR "population stratification"[ti] OR "population stratif*"[ti] OR "population segmentation"[ti] OR "population segment*"[ti] OR "Population Health Management"[majr] OR "population health management"[ti] OR "population health manag*"[ti] OR "empanelment"[ti] OR "empanelment*"[ti] OR "panel management"[ti] OR "panel management*"[ti])) OR (("tool"[ti] OR "tools"[ti] OR "intervention"[ti] OR "interventions"[ti]) AND ("risk stratification"[ti] OR "risk stratif*"[ti] OR "population stratification"[ti] OR "population stratif*"[ti] OR "population segmentation"[ti] OR "population segment*"[ti] OR "population health management"[ti] OR "population health manag*"[ti] OR "empanelment"[ti] OR "empanelment*"[ti] OR "panel management"[ti] OR "panel management*"[ti] OR "population-based approach"[ti] OR "population-based"[ti] OR "population panel"[ti] OR "population care"[ti]))))**)** AND ("Primary Health Care"[Mesh] OR "Primary Health Care"[all fields] OR "Primary Healthcare"[all fields] OR "Primary Care"[all fields] OR "Public Health"[ad] OR "Population Health"[ad] OR "General Practice"[Mesh] OR "General Practitioners"[Mesh] OR "General Practice"[tw] OR "General Practitioner"[tw] OR "General Practitioners"[tw] OR "Family Practice"[tw] OR "Family Practitioner"[tw] OR "Family Practitioners"[tw] OR "Physicians, Family"[Mesh] OR "Family Physician"[tw] OR "Family Physicians"[tw] OR "Family Doctor"[tw] OR "Family Doctors"[tw] OR "Primary Care Physician"[tw] OR "Primary Care Physicians"[tw]))

**MEDLINE via OVID**

<http://gateway.ovid.com/ovidweb.cgi?T=JS&MODE=ovid&NEWS=n&PAGE=main&D=medall>

(**(**(((exp "Cardiovascular Diseases"/ OR exp "Stroke"/ OR exp "Myocardial Infarction"/ OR exp "Brain Infarction"/ OR exp "Diabetes Mellitus"/ OR "cardiometabolic disease".mp OR "cardiometabolic diseases".mp OR "cardio-metabolic disease".mp OR "cardio-metabolic diseases".mp OR "cardiovascular disease".mp OR "cardiovascular diseases".mp OR "heart disease".mp OR "heart diseases".mp OR "stroke".mp OR "post-stroke".mp OR "TIA".mp OR "TIAs".mp OR "cerebrovascular accident".mp OR "cerebrovascular accident".mp OR "CBA".mp OR "CVAs".mp OR "myocardial infarction".mp OR "myocardial infarctions".mp OR "myocardial infarct*".mp OR "post-myocardial infarction".mp OR "brain infarction".mp OR "diabetes mellitus".mp OR "Diabetes".mp OR "IDDM".mp OR "NIDDM".mp OR "hypertension".mp) NOT ((exp "Infant"/ OR exp "Child"/ OR exp "Adolescent"/ OR "Infant".ti OR "Child".ti OR "Adolescent".ti OR "Infants".ti OR "Children".ti OR "Adolescents".ti) NOT (exp "Adult"/ OR "adult".mp OR "adults".ti OR "elderly".ti)) AND ("Risk"/ OR "risk".mp OR "risks".mp OR "atrisk".mp OR "outcome".mp OR "outcomes".mp)) AND ("risk stratification tools".ti OR "risk stratification tool".ti OR "population stratification tool".ti OR "population stratification tools".ti OR "population segmentation tools".ti OR "population segmentation tool".ti OR "population health management intervention".ti OR "population health management interventions".ti OR "empanelment intervention".ti OR "panel management intervention".ti OR "empanelment interventions".ti OR "panel management interventions".ti OR "risk stratification".ti OR "risk stratif*".ti OR "population stratification".ti OR "population stratif*".ti OR "population segmentation".ti OR "population segment*".ti OR exp *"Population Health Management"/ OR "population health management".ti OR "population health manag*".ti OR "empanelment".ti OR "empanelment*".ti OR "panel management".ti OR "panel management*".ti OR "population-based approach".ti OR "population-based".ti OR "population panel".ti OR "population care".ti) AND (exp "Pilot Projects"/ OR "Pilot Projects".mp OR "Pilot Project".mp OR "pilot".mp OR "implemented".mp OR "implementation".mp OR "implement*".mp OR "practice use".mp OR "practice uses".mp OR "clinical practice".mp OR "MDR application".mp OR "MDR applications".mp)) **OR** (((exp *"Cardiovascular Diseases"/ OR exp *"Stroke"/ OR exp *"Myocardial Infarction"/ OR exp *"Brain Infarction"/ OR exp *"Diabetes Mellitus"/ OR "cardiometabolic disease".ti OR "cardiometabolic diseases".ti OR "cardio-metabolic disease".ti OR "cardio-metabolic diseases".ti OR "cardiovascular disease".ti OR "cardiovascular diseases".ti OR "heart disease".ti OR "heart diseases".ti OR "stroke".ti OR "post-stroke".ti OR "TIA".ti OR "TIAs".ti OR "cerebrovascular accident".ti OR "cerebrovascular accident".ti OR "CBA".ti OR "CVAs".ti OR "myocardial infarction".ti OR "myocardial infarctions".ti OR "myocardial infarct*".ti OR "post-myocardial infarction".ti OR "brain infarction".ti OR "diabetes mellitus".ti OR "Diabetes".ti OR "IDDM".ti OR "NIDDM".ti OR "hypertension".ti) NOT ((exp "Infant"/ OR exp "Child"/ OR exp "Adolescent"/ OR "Infant".ti OR "Child".ti OR "Adolescent".ti OR "Infants".ti OR "Children".ti OR "Adolescents".ti) NOT (exp "Adult"/ OR "adult".mp OR "adults".ti OR "elderly".ti)) AND (exp "Risk"/ OR "risk".mp OR "risks".mp OR "atrisk".mp OR "outcome".mp OR "outcomes".mp)) AND ("risk stratification tools".mp OR "risk stratification tool".mp OR "population stratification tool".mp OR "population stratification tools".mp OR "population segmentation tools".mp OR "population segmentation tool".mp OR "population health management intervention".mp OR "population health management interventions".mp OR "empanelment intervention".mp OR "panel management intervention".mp OR "empanelment interventions".mp OR "panel management interventions".mp OR "risk stratification".mp OR "risk stratif*".mp OR "population stratification".mp OR "population stratif*".mp OR "population segmentation".mp OR "population segment*".mp OR exp "Population Health Management"/ OR "population health management".mp OR "population health manag*".mp OR "empanelment".mp OR "empanelment*".mp OR "panel management".mp OR "panel management*".mp OR "population-based approach".mp OR "population-based".mp OR "population panel".mp OR "population care".mp) AND (exp "Pilot Projects"/ OR "Pilot Projects".mp OR "Pilot Project".mp OR "pilot".mp OR "implemented".mp OR "implementation".mp OR "implement*".mp OR "practice use".mp OR "practice uses".mp OR "clinical practice".mp OR "MDR application".mp OR "MDR applications".mp)) **OR** (((exp *"Cardiovascular Diseases"/ OR exp *"Stroke"/ OR exp *"Myocardial Infarction"/ OR exp *"Brain Infarction"/ OR exp *"Diabetes Mellitus"/ OR "cardiometabolic disease".ti OR "cardiometabolic diseases".ti OR "cardio-metabolic disease".ti OR "cardio-metabolic diseases".ti OR "cardiovascular disease".ti OR "cardiovascular diseases".ti OR "heart disease".ti OR "heart diseases".ti OR "stroke".ti OR "post-stroke".ti OR "TIA".ti OR "TIAs".ti OR "cerebrovascular accident".ti OR "cerebrovascular accident".ti OR "CBA".ti OR "CVAs".ti OR "myocardial infarction".ti OR "myocardial infarctions".ti OR "myocardial infarct*".ti OR "post-myocardial infarction".ti OR "brain infarction".ti OR "diabetes mellitus".ti OR "Diabetes".ti OR "IDDM".ti OR "NIDDM".ti OR "hypertension".ti) NOT ((exp "Infant"/ OR exp "Child"/ OR exp "Adolescent"/ OR "Infant".ti OR "Child".ti OR "Adolescent".ti OR "Infants".ti OR "Children".ti OR "Adolescents".ti) NOT (exp "Adult"/ OR "adult".ti OR "adults".ti OR "elderly".ti)) AND (exp "Risk"/ OR "risk".mp OR "risks".mp OR "atrisk".mp OR "outcome".mp OR "outcomes".mp)) AND ("risk stratification tools".ti OR "risk stratification tool".ti OR "population stratification tool".ti OR "population stratification tools".ti OR "population segmentation tools".ti OR "population segmentation tool".ti OR "population health management intervention".ti OR "population health management interventions".ti OR "empanelment intervention".ti OR "panel management intervention".ti OR "empanelment interventions".ti OR "panel management interventions".ti OR (("risk stratification tools".mp OR "risk stratification tool".mp OR "population stratification tool".mp OR "population stratification tools".mp OR "population segmentation tools".mp OR "population segmentation tool".mp OR "population health management intervention".mp OR "population health management interventions".mp OR "empanelment intervention".mp OR "panel management intervention".mp OR "empanelment interventions".mp OR "panel management interventions".mp) AND ("risk stratification".ti OR "risk stratif*".ti OR "population stratification".ti OR "population stratif*".ti OR "population segmentation".ti OR "population segment*".ti OR exp *"Population Health Management"/ OR "population health management".ti OR "population health manag*".ti OR "empanelment".ti OR "empanelment*".ti OR "panel management".ti OR "panel management*".ti)) OR (("tool".ti OR "tools".ti OR "intervention".ti OR "interventions".ti) AND ("risk stratification".ti OR "risk stratif*".ti OR "population stratification".ti OR "population stratif*".ti OR "population segmentation".ti OR "population segment*".ti OR "population health management".ti OR "population health manag*".ti OR "empanelment".ti OR "empanelment*".ti OR "panel management".ti OR "panel management*".ti OR "population-based approach".ti OR "population-based".ti OR "population panel".ti OR "population care".ti))))**)** AND (exp "Primary Health Care"/ OR "Primary Health Care".af OR "Primary Healthcare".af OR "Primary Care".af OR "Public Health".in OR "Population Health".in OR exp "General Practice"/ OR exp "General Practitioners"/ OR "General Practice".mp OR "General Practitioner".mp OR "General Practitioners".mp OR "Family Practice".mp OR "Family Practitioner".mp OR "Family Practitioners".mp OR exp "Physicians, Family"/ OR "Family Physician".mp OR "Family Physicians".mp OR "Family Doctor".mp OR "Family Doctors".mp OR "Primary Care Physician".mp OR "Primary Care Physicians".mp))

**Embase**

<http://ovidsp.ovid.com/ovidweb.cgi?T=JS&PAGE=main&MODE=ovid&D=oemezd>

(**(**(((exp *"Cardiovascular Disease"/ OR exp *"cerebrovascular accident"/ OR exp *"Heart Infarction"/ OR exp *"Brain Infarction"/ OR exp *"Diabetes Mellitus"/ OR "cardiometabolic disease".ti,ab OR "cardiometabolic diseases".ti,ab OR "cardio-metabolic disease".ti,ab OR "cardio-metabolic diseases".ti,ab OR "cardiovascular disease".ti,ab OR "cardiovascular diseases".ti,ab OR "heart disease".ti,ab OR "heart diseases".ti,ab OR "stroke".ti,ab OR "post-stroke".ti,ab OR "TIA".ti,ab OR "TIAs".ti,ab OR "cerebrovascular accident".ti,ab OR "cerebrovascular accident".ti,ab OR "CBA".ti,ab OR "CVAs".ti,ab OR "myocardial infarction".ti,ab OR "myocardial infarctions".ti,ab OR "myocardial infarct*".ti,ab OR "post-myocardial infarction".ti,ab OR "brain infarction".ti,ab OR "diabetes mellitus".ti,ab OR "Diabetes".ti,ab OR "IDDM".ti,ab OR "NIDDM".ti,ab OR "hypertension".ti,ab) NOT ((exp "Infant"/ OR exp "Child"/ OR exp "Adolescent"/ OR "Infant".ti OR "Child".ti OR "Adolescent".ti OR "Infants".ti OR "Children".ti OR "Adolescents".ti) NOT (exp "Adult"/ OR "adult".ti,ab OR "adults".ti OR "elderly".ti)) AND ("Risk"/ OR "risk".ti,ab OR "risks".ti,ab OR "atrisk".ti,ab OR "outcome".ti,ab OR "outcomes".ti,ab)) AND ("risk stratification tools".ti OR "risk stratification tool".ti OR "population stratification tool".ti OR "population stratification tools".ti OR "population segmentation tools".ti OR "population segmentation tool".ti OR "population health management intervention".ti OR "population health management interventions".ti OR "empanelment intervention".ti OR "panel management intervention".ti OR "empanelment interventions".ti OR "panel management interventions".ti OR "risk stratification".ti OR "risk stratif*".ti OR "population stratification".ti OR "population stratif*".ti OR "population segmentation".ti OR "population segment*".ti OR exp *"Population Health Management"/ OR "population health management".ti OR "population health manag*".ti OR "empanelment".ti OR "empanelment*".ti OR "panel management".ti OR "panel management*".ti OR "population-based approach".ti OR "population-based".ti OR "population panel".ti OR "population care".ti) AND (exp "Pilot Study"/ OR "Pilot Projects".ti,ab OR "Pilot Project".ti,ab OR "pilot".ti,ab OR "implemented".ti,ab OR "implementation".ti,ab OR "implement*".ti,ab OR "practice use".ti,ab OR "practice uses".ti,ab OR "clinical practice".ti,ab OR "MDR application".ti,ab OR "MDR applications".ti,ab)) **OR** (((exp *"Cardiovascular Disease"/ OR exp *"cerebrovascular accident"/ OR exp *"Heart Infarction"/ OR exp *"Brain Infarction"/ OR exp *"Diabetes Mellitus"/ OR "cardiometabolic disease".ti OR "cardiometabolic diseases".ti OR "cardio-metabolic disease".ti OR "cardio-metabolic diseases".ti OR "cardiovascular disease".ti OR "cardiovascular diseases".ti OR "heart disease".ti OR "heart diseases".ti OR "stroke".ti OR "post-stroke".ti OR "TIA".ti OR "TIAs".ti OR "cerebrovascular accident".ti OR "cerebrovascular accident".ti OR "CBA".ti OR "CVAs".ti OR "myocardial infarction".ti OR "myocardial infarctions".ti OR "myocardial infarct*".ti OR "post-myocardial infarction".ti OR "brain infarction".ti OR "diabetes mellitus".ti OR "Diabetes".ti OR "IDDM".ti OR "NIDDM".ti OR "hypertension".ti) NOT ((exp "Infant"/ OR exp "Child"/ OR exp "Adolescent"/ OR "Infant".ti OR "Child".ti OR "Adolescent".ti OR "Infants".ti OR "Children".ti OR "Adolescents".ti) NOT (exp "Adult"/ OR "adult".ti,ab OR "adults".ti OR "elderly".ti)) AND (exp "Risk"/ OR "risk".ti,ab OR "risks".ti,ab OR "atrisk".ti,ab OR "outcome".ti,ab OR "outcomes".ti,ab)) AND ("risk stratification tools".ti,ab OR "risk stratification tool".ti,ab OR "population stratification tool".ti,ab OR "population stratification tools".ti,ab OR "population segmentation tools".ti,ab OR "population segmentation tool".ti,ab OR "population health management intervention".ti,ab OR "population health management interventions".ti,ab OR "empanelment intervention".ti,ab OR "panel management intervention".ti,ab OR "empanelment interventions".ti,ab OR "panel management interventions".ti,ab OR "risk stratification".ti,ab OR "risk stratif*".ti,ab OR "population stratification".ti,ab OR "population stratif*".ti,ab OR "population segmentation".ti,ab OR "population segment*".ti,ab OR exp "Population Health Management"/ OR "population health management".ti,ab OR "population health manag*".ti,ab OR "empanelment".ti,ab OR "empanelment*".ti,ab OR "panel management".ti,ab OR "panel management*".ti,ab OR "population-based approach".ti,ab OR "population-based".ti,ab OR "population panel".ti,ab OR "population care".ti,ab) AND (exp "Pilot Study"/ OR "Pilot Projects".ti,ab OR "Pilot Project".ti,ab OR "pilot".ti,ab OR "implemented".ti,ab OR "implementation".ti,ab OR "implement*".ti,ab OR "practice use".ti,ab OR "practice uses".ti,ab OR "clinical practice".ti,ab OR "MDR application".ti,ab OR "MDR applications".ti,ab)) **OR** (((exp *"Cardiovascular Disease"/ OR exp *"cerebrovascular accident"/ OR exp *"Heart Infarction"/ OR exp *"Brain Infarction"/ OR exp *"Diabetes Mellitus"/ OR "cardiometabolic disease".ti OR "cardiometabolic diseases".ti OR "cardio-metabolic disease".ti OR "cardio-metabolic diseases".ti OR "cardiovascular disease".ti OR "cardiovascular diseases".ti OR "heart disease".ti OR "heart diseases".ti OR "stroke".ti OR "post-stroke".ti OR "TIA".ti OR "TIAs".ti OR "cerebrovascular accident".ti OR "cerebrovascular accident".ti OR "CBA".ti OR "CVAs".ti OR "myocardial infarction".ti OR "myocardial infarctions".ti OR "myocardial infarct*".ti OR "post-myocardial infarction".ti OR "brain infarction".ti OR "diabetes mellitus".ti OR "Diabetes".ti OR "IDDM".ti OR "NIDDM".ti OR "hypertension".ti) NOT ((exp "Infant"/ OR exp "Child"/ OR exp "Adolescent"/ OR "Infant".ti OR "Child".ti OR "Adolescent".ti OR "Infants".ti OR "Children".ti OR "Adolescents".ti) NOT (exp "Adult"/ OR "adult".ti OR "adults".ti OR "elderly".ti)) AND (exp "Risk"/ OR "risk".ti,ab OR "risks".ti,ab OR "atrisk".ti,ab OR "outcome".ti,ab OR "outcomes".ti,ab)) AND ("risk stratification tools".ti OR "risk stratification tool".ti OR "population stratification tool".ti OR "population stratification tools".ti OR "population segmentation tools".ti OR "population segmentation tool".ti OR "population health management intervention".ti OR "population health management interventions".ti OR "empanelment intervention".ti OR "panel management intervention".ti OR "empanelment interventions".ti OR "panel management interventions".ti OR (("risk stratification tools".ti,ab OR "risk stratification tool".ti,ab OR "population stratification tool".ti,ab OR "population stratification tools".ti,ab OR "population segmentation tools".ti,ab OR "population segmentation tool".ti,ab OR "population health management intervention".ti,ab OR "population health management interventions".ti,ab OR "empanelment intervention".ti,ab OR "panel management intervention".ti,ab OR "empanelment interventions".ti,ab OR "panel management interventions".ti,ab) AND ("risk stratification".ti OR "risk stratif*".ti OR "population stratification".ti OR "population stratif*".ti OR "population segmentation".ti OR "population segment*".ti OR exp *"Population Health Management"/ OR "population health management".ti OR "population health manag*".ti OR "empanelment".ti OR "empanelment*".ti OR "panel management".ti OR "panel management*".ti)) OR (("tool".ti OR "tools".ti OR "intervention".ti OR "interventions".ti) AND ("risk stratification".ti OR "risk stratif*".ti OR "population stratification".ti OR "population stratif*".ti OR "population segmentation".ti OR "population segment*".ti OR "population health management".ti OR "population health manag*".ti OR "empanelment".ti OR "empanelment*".ti OR "panel management".ti OR "panel management*".ti OR "population-based approach".ti OR "population-based".ti OR "population panel".ti OR "population care".ti))))**)** AND (exp "Primary Health Care"/ OR "Primary Health Care".af OR "Primary Healthcare".af OR "Primary Care".af OR "Public Health".in OR "Population Health".in OR exp "General Practice"/ OR exp "General Practitioner"/ OR "General Practice".ti,ab OR "General Practitioner".ti,ab OR "General Practitioners".ti,ab OR "Family Practice".ti,ab OR "Family Practitioner".ti,ab OR "Family Practitioners".ti,ab OR "Family Physician".ti,ab OR "Family Physicians".ti,ab OR "Family Doctor".ti,ab OR "Family Doctors".ti,ab OR "Primary Care Physician".ti,ab OR "Primary Care Physicians".ti,ab)) NOT (conference review or conference abstract).pt

**Web of Science**

<http://isiknowledge.com/wos>

(**(**((TS=("Cardiovascular Disease" OR "cerebrovascular accident" OR "Heart Infarction" OR "Brain Infarction" OR "Diabetes Mellitus" OR "cardiometabolic disease" OR "cardiometabolic diseases" OR "cardio-metabolic disease" OR "cardio-metabolic diseases" OR "cardiovascular disease" OR "cardiovascular diseases" OR "heart disease" OR "heart diseases" OR "stroke" OR "post-stroke" OR "TIA" OR "TIAs" OR "cerebrovascular accident" OR "cerebrovascular accident" OR "CBA" OR "CVAs" OR "myocardial infarction" OR "myocardial infarctions" OR "myocardial infarct*" OR "post-myocardial infarction" OR "brain infarction" OR "diabetes mellitus" OR "Diabetes" OR "IDDM" OR "NIDDM" OR "hypertension") NOT TI=((exp "Infant" OR exp "Child" OR exp "Adolescent" OR "Infant" OR "Child" OR "Adolescent" OR "Infants" OR "Children" OR "Adolescents") NOT (exp "Adult" OR "adult" OR "adults" OR "elderly")) AND TS=("Risk" OR "risk" OR "risks" OR "atrisk" OR "outcome" OR "outcomes")) AND TI=("risk stratification tools" OR "risk stratification tool" OR "population stratification tool" OR "population stratification tools" OR "population segmentation tools" OR "population segmentation tool" OR "population health management intervention" OR "population health management interventions" OR "empanelment intervention" OR "panel management intervention" OR "empanelment interventions" OR "panel management interventions" OR "risk stratification" OR "risk stratif*" OR "population stratification" OR "population stratif*" OR "population segmentation" OR "population segment*" OR "Population Health Management" OR "population health management" OR "population health manag*" OR "empanelment" OR "empanelment*" OR "panel management" OR "panel management*" OR "population-based approach" OR "population-based" OR "population panel" OR "population care") AND TS=(exp "Pilot Study" OR "Pilot Projects" OR "Pilot Project" OR "pilot" OR "implemented" OR "implementation" OR "implement*" OR "practice use" OR "practice uses" OR "clinical practice" OR "MDR application" OR "MDR applications")) **OR** ((TI=("Cardiovascular Disease" OR "cerebrovascular accident" OR "Heart Infarction" OR "Brain Infarction" OR "Diabetes Mellitus" OR "cardiometabolic disease" OR "cardiometabolic diseases" OR "cardio-metabolic disease" OR "cardio-metabolic diseases" OR "cardiovascular disease" OR "cardiovascular diseases" OR "heart disease" OR "heart diseases" OR "stroke" OR "post-stroke" OR "TIA" OR "TIAs" OR "cerebrovascular accident" OR "cerebrovascular accident" OR "CBA" OR "CVAs" OR "myocardial infarction" OR "myocardial infarctions" OR "myocardial infarct*" OR "post-myocardial infarction" OR "brain infarction" OR "diabetes mellitus" OR "Diabetes" OR "IDDM" OR "NIDDM" OR "hypertension") NOT TI=((exp "Infant" OR exp "Child" OR exp "Adolescent" OR "Infant" OR "Child" OR "Adolescent" OR "Infants" OR "Children" OR "Adolescents") NOT (exp "Adult" OR "adult" OR "adults" OR "elderly")) AND TS=(exp "Risk" OR "risk" OR "risks" OR "atrisk" OR "outcome" OR "outcomes")) AND TS=("risk stratification tools" OR "risk stratification tool" OR "population stratification tool" OR "population stratification tools" OR "population segmentation tools" OR "population segmentation tool" OR "population health management intervention" OR "population health management interventions" OR "empanelment intervention" OR "panel management intervention" OR "empanelment interventions" OR "panel management interventions" OR "risk stratification" OR "risk stratif*" OR "population stratification" OR "population stratif*" OR "population segmentation" OR "population segment*" OR exp "Population Health Management" OR "population health management" OR "population health manag*" OR "empanelment" OR "empanelment*" OR "panel management" OR "panel management*" OR "population-based approach" OR "population-based" OR "population panel" OR "population care") AND TS=(exp "Pilot Study" OR "Pilot Projects" OR "Pilot Project" OR "pilot" OR "implemented" OR "implementation" OR "implement*" OR "practice use" OR "practice uses" OR "clinical practice" OR "MDR application" OR "MDR applications")) **OR** ((TI=("Cardiovascular Disease" OR "cerebrovascular accident" OR "Heart Infarction" OR "Brain Infarction" OR "Diabetes Mellitus" OR "cardiometabolic disease" OR "cardiometabolic diseases" OR "cardio-metabolic disease" OR "cardio-metabolic diseases" OR "cardiovascular disease" OR "cardiovascular diseases" OR "heart disease" OR "heart diseases" OR "stroke" OR "post-stroke" OR "TIA" OR "TIAs" OR "cerebrovascular accident" OR "cerebrovascular accident" OR "CBA" OR "CVAs" OR "myocardial infarction" OR "myocardial infarctions" OR "myocardial infarct*" OR "post-myocardial infarction" OR "brain infarction" OR "diabetes mellitus" OR "Diabetes" OR "IDDM" OR "NIDDM" OR "hypertension") NOT TI=((exp "Infant" OR exp "Child" OR exp "Adolescent" OR "Infant" OR "Child" OR "Adolescent" OR "Infants" OR "Children" OR "Adolescents") NOT (exp "Adult" OR "adult" OR "adults" OR "elderly")) AND TS=(exp "Risk" OR "risk" OR "risks" OR "atrisk" OR "outcome" OR "outcomes")) AND TI=("risk stratification tools" OR "risk stratification tool" OR "population stratification tool" OR "population stratification tools" OR "population segmentation tools" OR "population segmentation tool" OR "population health management intervention" OR "population health management interventions" OR "empanelment intervention" OR "panel management intervention" OR "empanelment interventions" OR "panel management interventions" OR (("risk stratification tools" OR "risk stratification tool" OR "population stratification tool" OR "population stratification tools" OR "population segmentation tools" OR "population segmentation tool" OR "population health management intervention" OR "population health management interventions" OR "empanelment intervention" OR "panel management intervention" OR "empanelment interventions" OR "panel management interventions") AND ("risk stratification" OR "risk stratif*" OR "population stratification" OR "population stratif*" OR "population segmentation" OR "population segment*" OR "Population Health Management" OR "population health management" OR "population health manag*" OR "empanelment" OR "empanelment*" OR "panel management" OR "panel management*")) OR (("tool" OR "tools" OR "intervention" OR "interventions") AND ("risk stratification" OR "risk stratif*" OR "population stratification" OR "population stratif*" OR "population segmentation" OR "population segment*" OR "population health management" OR "population health manag*" OR "empanelment" OR "empanelment*" OR "panel management" OR "panel management*" OR "population-based approach" OR "population-based" OR "population panel" OR "population care"))))**)** AND TS=(exp "Primary Health Care" OR "Primary Health Care" OR "Primary Healthcare" OR "Primary Care" OR "Public Health" OR "Population Health" OR exp "General Practice" OR exp "General Practitioner" OR "General Practice" OR "General Practitioner" OR "General Practitioners" OR "Family Practice" OR "Family Practitioner" OR "Family Practitioners" OR "Family Physician" OR "Family Physicians" OR "Family Doctor" OR "Family Doctors" OR "Primary Care Physician" OR "Primary Care Physicians")) NOT DT=(meeting abstract)

**Cochrane**

<https://www.cochranelibrary.com/advanced-search/search-manager>

(**(**((("Cardiovascular Disease" OR "cerebrovascular accident" OR "Heart Infarction" OR "Brain Infarction" OR "Diabetes Mellitus" OR "cardiometabolic disease" OR "cardiometabolic diseases" OR "cardio metabolic disease" OR "cardio metabolic diseases" OR "cardiovascular disease" OR "cardiovascular diseases" OR "heart disease" OR "heart diseases" OR "stroke" OR "post stroke" OR "TIA" OR "TIAs" OR "cerebrovascular accident" OR "cerebrovascular accident" OR "CBA" OR "CVAs" OR "myocardial infarction" OR "myocardial infarctions" OR "myocardial infarct*" OR "post myocardial infarction" OR "brain infarction" OR "diabetes mellitus" OR "Diabetes" OR "IDDM" OR "NIDDM" OR "hypertension") NOT ((exp "Infant" OR exp "Child" OR exp "Adolescent" OR "Infant" OR "Child" OR "Adolescent" OR "Infants" OR "Children" OR "Adolescents") NOT (exp "Adult" OR "adult" OR "adults" OR "elderly")) AND ("Risk" OR "risk" OR "risks" OR "atrisk" OR "outcome" OR "outcomes")):ti,ab,kw AND ("risk stratification tools" OR "risk stratification tool" OR "population stratification tool" OR "population stratification tools" OR "population segmentation tools" OR "population segmentation tool" OR "population health management intervention" OR "population health management interventions" OR "empanelment intervention" OR "panel management intervention" OR "empanelment interventions" OR "panel management interventions" OR "risk stratification" OR "risk stratif*" OR "population stratification" OR "population stratif*" OR "population segmentation" OR "population segment*" OR "Population Health Management" OR "population health management" OR "population health manag*" OR "empanelment" OR "empanelment*" OR "panel management" OR "panel management*" OR "population based approach" OR "population based" OR "population panel" OR "population care"):ti AND (exp "Pilot Study" OR "Pilot Projects" OR "Pilot Project" OR "pilot" OR "implemented" OR "implementation" OR "implement*" OR "practice use" OR "practice uses" OR "clinical practice" OR "MDR application" OR "MDR applications"):ti,ab,kw) **OR** ((("Cardiovascular Disease" OR "cerebrovascular accident" OR "Heart Infarction" OR "Brain Infarction" OR "Diabetes Mellitus" OR "cardiometabolic disease" OR "cardiometabolic diseases" OR "cardio metabolic disease" OR "cardio metabolic diseases" OR "cardiovascular disease" OR "cardiovascular diseases" OR "heart disease" OR "heart diseases" OR "stroke" OR "post stroke" OR "TIA" OR "TIAs" OR "cerebrovascular accident" OR "cerebrovascular accident" OR "CBA" OR "CVAs" OR "myocardial infarction" OR "myocardial infarctions" OR "myocardial infarct*" OR "post myocardial infarction" OR "brain infarction" OR "diabetes mellitus" OR "Diabetes" OR "IDDM" OR "NIDDM" OR "hypertension"):ti NOT ((exp "Infant" OR exp "Child" OR exp "Adolescent" OR "Infant" OR "Child" OR "Adolescent" OR "Infants" OR "Children" OR "Adolescents") NOT (exp "Adult" OR "adult" OR "adults" OR "elderly")):ti AND (exp "Risk" OR "risk" OR "risks" OR "atrisk" OR "outcome" OR "outcomes"):ti,ab,kw) AND ("risk stratification tools" OR "risk stratification tool" OR "population stratification tool" OR "population stratification tools" OR "population segmentation tools" OR "population segmentation tool" OR "population health management intervention" OR "population health management interventions" OR "empanelment intervention" OR "panel management intervention" OR "empanelment interventions" OR "panel management interventions" OR "risk stratification" OR "risk stratif*" OR "population stratification" OR "population stratif*" OR "population segmentation" OR "population segment*" OR exp "Population Health Management" OR "population health management" OR "population health manag*" OR "empanelment" OR "empanelment*" OR "panel management" OR "panel management*" OR "population based approach" OR "population based" OR "population panel" OR "population care"):ti,ab,kw AND (exp "Pilot Study" OR "Pilot Projects" OR "Pilot Project" OR "pilot" OR "implemented" OR "implementation" OR "implement*" OR "practice use" OR "practice uses" OR "clinical practice" OR "MDR application" OR "MDR applications"):ti,ab,kw) **OR** ((("Cardiovascular Disease" OR "cerebrovascular accident" OR "Heart Infarction" OR "Brain Infarction" OR "Diabetes Mellitus" OR "cardiometabolic disease" OR "cardiometabolic diseases" OR "cardio metabolic disease" OR "cardio metabolic diseases" OR "cardiovascular disease" OR "cardiovascular diseases" OR "heart disease" OR "heart diseases" OR "stroke" OR "post stroke" OR "TIA" OR "TIAs" OR "cerebrovascular accident" OR "cerebrovascular accident" OR "CBA" OR "CVAs" OR "myocardial infarction" OR "myocardial infarctions" OR "myocardial infarct*" OR "post myocardial infarction" OR "brain infarction" OR "diabetes mellitus" OR "Diabetes" OR "IDDM" OR "NIDDM" OR "hypertension"):ti NOT ((exp "Infant" OR exp "Child" OR exp "Adolescent" OR "Infant" OR "Child" OR "Adolescent" OR "Infants" OR "Children" OR "Adolescents") NOT (exp "Adult" OR "adult" OR "adults" OR "elderly")):ti AND (exp "Risk" OR "risk" OR "risks" OR "atrisk" OR "outcome" OR "outcomes"):ti,ab,kw) AND ("risk stratification tools" OR "risk stratification tool" OR "population stratification tool" OR "population stratification tools" OR "population segmentation tools" OR "population segmentation tool" OR "population health management intervention" OR "population health management interventions" OR "empanelment intervention" OR "panel management intervention" OR "empanelment interventions" OR "panel management interventions" OR (("risk stratification tools" OR "risk stratification tool" OR "population stratification tool" OR "population stratification tools" OR "population segmentation tools" OR "population segmentation tool" OR "population health management intervention" OR "population health management interventions" OR "empanelment intervention" OR "panel management intervention" OR "empanelment interventions" OR "panel management interventions") AND ("risk stratification" OR "risk stratif*" OR "population stratification" OR "population stratif*" OR "population segmentation" OR "population segment*" OR "Population Health Management" OR "population health management" OR "population health manag*" OR "empanelment" OR "empanelment*" OR "panel management" OR "panel management*")) OR (("tool" OR "tools" OR "intervention" OR "interventions") AND ("risk stratification" OR "risk stratif*" OR "population stratification" OR "population stratif*" OR "population segmentation" OR "population segment*" OR "population health management" OR "population health manag*" OR "empanelment" OR "empanelment*" OR "panel management" OR "panel management*" OR "population based approach" OR "population based" OR "population panel" OR "population care"))):ti)**)** AND (exp "Primary Health Care" OR "Primary Health Care" OR "Primary Healthcare" OR "Primary Care" OR "Public Health" OR "Population Health" OR exp "General Practice" OR exp "General Practitioner" OR "General Practice" OR "General Practitioner" OR "General Practitioners" OR "Family Practice" OR "Family Practitioner" OR "Family Practitioners" OR "Family Physician" OR "Family Physicians" OR "Family Doctor" OR "Family Doctors" OR "Primary Care Physician" OR "Primary Care Physicians"):ti,ab,kw)

**Emcare** <http://ovidsp.ovid.com/ovidweb.cgi?T=JS&NEWS=n&CSC=Y&PAGE=main&D=emcr>

(**(**(((exp *"Cardiovascular Disease"/ OR exp *"cerebrovascular accident"/ OR exp *"Heart Infarction"/ OR exp *"Brain Infarction"/ OR exp *"Diabetes Mellitus"/ OR "cardiometabolic disease".ti,ab OR "cardiometabolic diseases".ti,ab OR "cardio-metabolic disease".ti,ab OR "cardio-metabolic diseases".ti,ab OR "cardiovascular disease".ti,ab OR "cardiovascular diseases".ti,ab OR "heart disease".ti,ab OR "heart diseases".ti,ab OR "stroke".ti,ab OR "post-stroke".ti,ab OR "TIA".ti,ab OR "TIAs".ti,ab OR "cerebrovascular accident".ti,ab OR "cerebrovascular accident".ti,ab OR "CBA".ti,ab OR "CVAs".ti,ab OR "myocardial infarction".ti,ab OR "myocardial infarctions".ti,ab OR "myocardial infarct*".ti,ab OR "post-myocardial infarction".ti,ab OR "brain infarction".ti,ab OR "diabetes mellitus".ti,ab OR "Diabetes".ti,ab OR "IDDM".ti,ab OR "NIDDM".ti,ab OR "hypertension".ti,ab) NOT ((exp "Infant"/ OR exp "Child"/ OR exp "Adolescent"/ OR "Infant".ti OR "Child".ti OR "Adolescent".ti OR "Infants".ti OR "Children".ti OR "Adolescents".ti) NOT (exp "Adult"/ OR "adult".ti,ab OR "adults".ti OR "elderly".ti)) AND ("Risk"/ OR "risk".ti,ab OR "risks".ti,ab OR "atrisk".ti,ab OR "outcome".ti,ab OR "outcomes".ti,ab)) AND ("risk stratification tools".ti OR "risk stratification tool".ti OR "population stratification tool".ti OR "population stratification tools".ti OR "population segmentation tools".ti OR "population segmentation tool".ti OR "population health management intervention".ti OR "population health management interventions".ti OR "empanelment intervention".ti OR "panel management intervention".ti OR "empanelment interventions".ti OR "panel management interventions".ti OR "risk stratification".ti OR "risk stratif*".ti OR "population stratification".ti OR "population stratif*".ti OR "population segmentation".ti OR "population segment*".ti OR exp *"Population Health Management"/ OR "population health management".ti OR "population health manag*".ti OR "empanelment".ti OR "empanelment*".ti OR "panel management".ti OR "panel management*".ti OR "population-based approach".ti OR "population-based".ti OR "population panel".ti OR "population care".ti) AND (exp "Pilot Study"/ OR "Pilot Projects".ti,ab OR "Pilot Project".ti,ab OR "pilot".ti,ab OR "implemented".ti,ab OR "implementation".ti,ab OR "implement*".ti,ab OR "practice use".ti,ab OR "practice uses".ti,ab OR "clinical practice".ti,ab OR "MDR application".ti,ab OR "MDR applications".ti,ab)) **OR** (((exp *"Cardiovascular Disease"/ OR exp *"cerebrovascular accident"/ OR exp *"Heart Infarction"/ OR exp *"Brain Infarction"/ OR exp *"Diabetes Mellitus"/ OR "cardiometabolic disease".ti OR "cardiometabolic diseases".ti OR "cardio-metabolic disease".ti OR "cardio-metabolic diseases".ti OR "cardiovascular disease".ti OR "cardiovascular diseases".ti OR "heart disease".ti OR "heart diseases".ti OR "stroke".ti OR "post-stroke".ti OR "TIA".ti OR "TIAs".ti OR "cerebrovascular accident".ti OR "cerebrovascular accident".ti OR "CBA".ti OR "CVAs".ti OR "myocardial infarction".ti OR "myocardial infarctions".ti OR "myocardial infarct*".ti OR "post-myocardial infarction".ti OR "brain infarction".ti OR "diabetes mellitus".ti OR "Diabetes".ti OR "IDDM".ti OR "NIDDM".ti OR "hypertension".ti) NOT ((exp "Infant"/ OR exp "Child"/ OR exp "Adolescent"/ OR "Infant".ti OR "Child".ti OR "Adolescent".ti OR "Infants".ti OR "Children".ti OR "Adolescents".ti) NOT (exp "Adult"/ OR "adult".ti,ab OR "adults".ti OR "elderly".ti)) AND (exp "Risk"/ OR "risk".ti,ab OR "risks".ti,ab OR "atrisk".ti,ab OR "outcome".ti,ab OR "outcomes".ti,ab)) AND ("risk stratification tools".ti,ab OR "risk stratification tool".ti,ab OR "population stratification tool".ti,ab OR "population stratification tools".ti,ab OR "population segmentation tools".ti,ab OR "population segmentation tool".ti,ab OR "population health management intervention".ti,ab OR "population health management interventions".ti,ab OR "empanelment intervention".ti,ab OR "panel management intervention".ti,ab OR "empanelment interventions".ti,ab OR "panel management interventions".ti,ab OR "risk stratification".ti,ab OR "risk stratif*".ti,ab OR "population stratification".ti,ab OR "population stratif*".ti,ab OR "population segmentation".ti,ab OR "population segment*".ti,ab OR exp "Population Health Management"/ OR "population health management".ti,ab OR "population health manag*".ti,ab OR "empanelment".ti,ab OR "empanelment*".ti,ab OR "panel management".ti,ab OR "panel management*".ti,ab OR "population-based approach".ti,ab OR "population-based".ti,ab OR "population panel".ti,ab OR "population care".ti,ab) AND (exp "Pilot Study"/ OR "Pilot Projects".ti,ab OR "Pilot Project".ti,ab OR "pilot".ti,ab OR "implemented".ti,ab OR "implementation".ti,ab OR "implement*".ti,ab OR "practice use".ti,ab OR "practice uses".ti,ab OR "clinical practice".ti,ab OR "MDR application".ti,ab OR "MDR applications".ti,ab)) **OR** (((exp *"Cardiovascular Disease"/ OR exp *"cerebrovascular accident"/ OR exp *"Heart Infarction"/ OR exp *"Brain Infarction"/ OR exp *"Diabetes Mellitus"/ OR "cardiometabolic disease".ti OR "cardiometabolic diseases".ti OR "cardio-metabolic disease".ti OR "cardio-metabolic diseases".ti OR "cardiovascular disease".ti OR "cardiovascular diseases".ti OR "heart disease".ti OR "heart diseases".ti OR "stroke".ti OR "post-stroke".ti OR "TIA".ti OR "TIAs".ti OR "cerebrovascular accident".ti OR "cerebrovascular accident".ti OR "CBA".ti OR "CVAs".ti OR "myocardial infarction".ti OR "myocardial infarctions".ti OR "myocardial infarct*".ti OR "post-myocardial infarction".ti OR "brain infarction".ti OR "diabetes mellitus".ti OR "Diabetes".ti OR "IDDM".ti OR "NIDDM".ti OR "hypertension".ti) NOT ((exp "Infant"/ OR exp "Child"/ OR exp "Adolescent"/ OR "Infant".ti OR "Child".ti OR "Adolescent".ti OR "Infants".ti OR "Children".ti OR "Adolescents".ti) NOT (exp "Adult"/ OR "adult".ti OR "adults".ti OR "elderly".ti)) AND (exp "Risk"/ OR "risk".ti,ab OR "risks".ti,ab OR "atrisk".ti,ab OR "outcome".ti,ab OR "outcomes".ti,ab)) AND ("risk stratification tools".ti OR "risk stratification tool".ti OR "population stratification tool".ti OR "population stratification tools".ti OR "population segmentation tools".ti OR "population segmentation tool".ti OR "population health management intervention".ti OR "population health management interventions".ti OR "empanelment intervention".ti OR "panel management intervention".ti OR "empanelment interventions".ti OR "panel management interventions".ti OR (("risk stratification tools".ti,ab OR "risk stratification tool".ti,ab OR "population stratification tool".ti,ab OR "population stratification tools".ti,ab OR "population segmentation tools".ti,ab OR "population segmentation tool".ti,ab OR "population health management intervention".ti,ab OR "population health management interventions".ti,ab OR "empanelment intervention".ti,ab OR "panel management intervention".ti,ab OR "empanelment interventions".ti,ab OR "panel management interventions".ti,ab) AND ("risk stratification".ti OR "risk stratif*".ti OR "population stratification".ti OR "population stratif*".ti OR "population segmentation".ti OR "population segment*".ti OR exp *"Population Health Management"/ OR "population health management".ti OR "population health manag*".ti OR "empanelment".ti OR "empanelment*".ti OR "panel management".ti OR "panel management*".ti)) OR (("tool".ti OR "tools".ti OR "intervention".ti OR "interventions".ti) AND ("risk stratification".ti OR "risk stratif*".ti OR "population stratification".ti OR "population stratif*".ti OR "population segmentation".ti OR "population segment*".ti OR "population health management".ti OR "population health manag*".ti OR "empanelment".ti OR "empanelment*".ti OR "panel management".ti OR "panel management*".ti OR "population-based approach".ti OR "population-based".ti OR "population panel".ti OR "population care".ti))))**)** AND (exp "Primary Health Care"/ OR "Primary Health Care".af OR "Primary Healthcare".af OR "Primary Care".af OR "Public Health".in OR "Population Health".in OR exp "General Practice"/ OR exp "General Practitioner"/ OR "General Practice".ti,ab OR "General Practitioner".ti,ab OR "General Practitioners".ti,ab OR "Family Practice".ti,ab OR "Family Practitioner".ti,ab OR "Family Practitioners".ti,ab OR "Family Physician".ti,ab OR "Family Physicians".ti,ab OR "Family Doctor".ti,ab OR "Family Doctors".ti,ab OR "Primary Care Physician".ti,ab OR "Primary Care Physicians".ti,ab))

**Academic Search Premier**

<http://search.ebscohost.com/login.aspx?authtype=ip,uid&profile=lumc&defaultdb=aph>

(**(**(TI(("Cardiovascular Disease" OR "cerebrovascular accident" OR "Heart Infarction" OR "Brain Infarction" OR "Diabetes Mellitus" OR "cardiometabolic disease" OR "cardiometabolic diseases" OR "cardio-metabolic disease" OR "cardio-metabolic diseases" OR "cardiovascular disease" OR "cardiovascular diseases" OR "heart disease" OR "heart diseases" OR "stroke" OR "post-stroke" OR "TIA" OR "TIAs" OR "cerebrovascular accident" OR "cerebrovascular accident" OR "CBA" OR "CVAs" OR "myocardial infarction" OR "myocardial infarctions" OR "myocardial infarct*" OR "post-myocardial infarction" OR "brain infarction" OR "diabetes mellitus" OR "Diabetes" OR "IDDM" OR "NIDDM" OR "hypertension") NOT ((exp "Infant" OR exp "Child" OR exp "Adolescent" OR "Infant" OR "Child" OR "Adolescent" OR "Infants" OR "Children" OR "Adolescents") NOT (exp "Adult" OR "adult" OR "adults" OR "elderly")) AND ("Risk" OR "risk" OR "risks" OR "atrisk" OR "outcome" OR "outcomes")) AND TI("risk stratification tools" OR "risk stratification tool" OR "population stratification tool" OR "population stratification tools" OR "population segmentation tools" OR "population segmentation tool" OR "population health management intervention" OR "population health management interventions" OR "empanelment intervention" OR "panel management intervention" OR "empanelment interventions" OR "panel management interventions" OR "risk stratification" OR "risk stratif*" OR "population stratification" OR "population stratif*" OR "population segmentation" OR "population segment*" OR "Population Health Management" OR "population health management" OR "population health manag*" OR "empanelment" OR "empanelment*" OR "panel management" OR "panel management*" OR "population-based approach" OR "population-based" OR "population panel" OR "population care") AND TI(exp "Pilot Study" OR "Pilot Projects" OR "Pilot Project" OR "pilot" OR "implemented" OR "implementation" OR "implement*" OR "practice use" OR "practice uses" OR "clinical practice" OR "MDR application" OR "MDR applications")) **OR** ((TI("Cardiovascular Disease" OR "cerebrovascular accident" OR "Heart Infarction" OR "Brain Infarction" OR "Diabetes Mellitus" OR "cardiometabolic disease" OR "cardiometabolic diseases" OR "cardio-metabolic disease" OR "cardio-metabolic diseases" OR "cardiovascular disease" OR "cardiovascular diseases" OR "heart disease" OR "heart diseases" OR "stroke" OR "post-stroke" OR "TIA" OR "TIAs" OR "cerebrovascular accident" OR "cerebrovascular accident" OR "CBA" OR "CVAs" OR "myocardial infarction" OR "myocardial infarctions" OR "myocardial infarct*" OR "post-myocardial infarction" OR "brain infarction" OR "diabetes mellitus" OR "Diabetes" OR "IDDM" OR "NIDDM" OR "hypertension") NOT TI((exp "Infant" OR exp "Child" OR exp "Adolescent" OR "Infant" OR "Child" OR "Adolescent" OR "Infants" OR "Children" OR "Adolescents") NOT (exp "Adult" OR "adult" OR "adults" OR "elderly")) AND TI(exp "Risk" OR "risk" OR "risks" OR "atrisk" OR "outcome" OR "outcomes")) AND TI("risk stratification tools" OR "risk stratification tool" OR "population stratification tool" OR "population stratification tools" OR "population segmentation tools" OR "population segmentation tool" OR "population health management intervention" OR "population health management interventions" OR "empanelment intervention" OR "panel management intervention" OR "empanelment interventions" OR "panel management interventions" OR "risk stratification" OR "risk stratif*" OR "population stratification" OR "population stratif*" OR "population segmentation" OR "population segment*" OR exp "Population Health Management" OR "population health management" OR "population health manag*" OR "empanelment" OR "empanelment*" OR "panel management" OR "panel management*" OR "population-based approach" OR "population-based" OR "population panel" OR "population care") AND TI(exp "Pilot Study" OR "Pilot Projects" OR "Pilot Project" OR "pilot" OR "implemented" OR "implementation" OR "implement*" OR "practice use" OR "practice uses" OR "clinical practice" OR "MDR application" OR "MDR applications")) **OR** ((TI("Cardiovascular Disease" OR "cerebrovascular accident" OR "Heart Infarction" OR "Brain Infarction" OR "Diabetes Mellitus" OR "cardiometabolic disease" OR "cardiometabolic diseases" OR "cardio-metabolic disease" OR "cardio-metabolic diseases" OR "cardiovascular disease" OR "cardiovascular diseases" OR "heart disease" OR "heart diseases" OR "stroke" OR "post-stroke" OR "TIA" OR "TIAs" OR "cerebrovascular accident" OR "cerebrovascular accident" OR "CBA" OR "CVAs" OR "myocardial infarction" OR "myocardial infarctions" OR "myocardial infarct*" OR "post-myocardial infarction" OR "brain infarction" OR "diabetes mellitus" OR "Diabetes" OR "IDDM" OR "NIDDM" OR "hypertension") NOT TI((exp "Infant" OR exp "Child" OR exp "Adolescent" OR "Infant" OR "Child" OR "Adolescent" OR "Infants" OR "Children" OR "Adolescents") NOT (exp "Adult" OR "adult" OR "adults" OR "elderly")) AND TI(exp "Risk" OR "risk" OR "risks" OR "atrisk" OR "outcome" OR "outcomes")) AND TI("risk stratification tools" OR "risk stratification tool" OR "population stratification tool" OR "population stratification tools" OR "population segmentation tools" OR "population segmentation tool" OR "population health management intervention" OR "population health management interventions" OR "empanelment intervention" OR "panel management intervention" OR "empanelment interventions" OR "panel management interventions" OR (("risk stratification tools" OR "risk stratification tool" OR "population stratification tool" OR "population stratification tools" OR "population segmentation tools" OR "population segmentation tool" OR "population health management intervention" OR "population health management interventions" OR "empanelment intervention" OR "panel management intervention" OR "empanelment interventions" OR "panel management interventions") AND ("risk stratification" OR "risk stratif*" OR "population stratification" OR "population stratif*" OR "population segmentation" OR "population segment*" OR "Population Health Management" OR "population health management" OR "population health manag*" OR "empanelment" OR "empanelment*" OR "panel management" OR "panel management*")) OR (("tool" OR "tools" OR "intervention" OR "interventions") AND ("risk stratification" OR "risk stratif*" OR "population stratification" OR "population stratif*" OR "population segmentation" OR "population segment*" OR "population health management" OR "population health manag*" OR "empanelment" OR "empanelment*" OR "panel management" OR "panel management*" OR "population-based approach" OR "population-based" OR "population panel" OR "population care"))))**)** AND TI(exp "Primary Health Care" OR "Primary Health Care" OR "Primary Healthcare" OR "Primary Care" OR "Public Health" OR "Population Health" OR exp "General Practice" OR exp "General Practitioner" OR "General Practice" OR "General Practitioner" OR "General Practitioners" OR "Family Practice" OR "Family Practitioner" OR "Family Practitioners" OR "Family Physician" OR "Family Physicians" OR "Family Doctor" OR "Family Doctors" OR "Primary Care Physician" OR "Primary Care Physicians"))

SU

(**(**(SU(("Cardiovascular Disease" OR "cerebrovascular accident" OR "Heart Infarction" OR "Brain Infarction" OR "Diabetes Mellitus" OR "cardiometabolic disease" OR "cardiometabolic diseases" OR "cardio-metabolic disease" OR "cardio-metabolic diseases" OR "cardiovascular disease" OR "cardiovascular diseases" OR "heart disease" OR "heart diseases" OR "stroke" OR "post-stroke" OR "TIA" OR "TIAs" OR "cerebrovascular accident" OR "cerebrovascular accident" OR "CBA" OR "CVAs" OR "myocardial infarction" OR "myocardial infarctions" OR "myocardial infarct*" OR "post-myocardial infarction" OR "brain infarction" OR "diabetes mellitus" OR "Diabetes" OR "IDDM" OR "NIDDM" OR "hypertension") NOT ((exp "Infant" OR exp "Child" OR exp "Adolescent" OR "Infant" OR "Child" OR "Adolescent" OR "Infants" OR "Children" OR "Adolescents") NOT (exp "Adult" OR "adult" OR "adults" OR "elderly")) AND ("Risk" OR "risk" OR "risks" OR "atrisk" OR "outcome" OR "outcomes")) AND TI("risk stratification tools" OR "risk stratification tool" OR "population stratification tool" OR "population stratification tools" OR "population segmentation tools" OR "population segmentation tool" OR "population health management intervention" OR "population health management interventions" OR "empanelment intervention" OR "panel management intervention" OR "empanelment interventions" OR "panel management interventions" OR "risk stratification" OR "risk stratif*" OR "population stratification" OR "population stratif*" OR "population segmentation" OR "population segment*" OR "Population Health Management" OR "population health management" OR "population health manag*" OR "empanelment" OR "empanelment*" OR "panel management" OR "panel management*" OR "population-based approach" OR "population-based" OR "population panel" OR "population care") AND SU(exp "Pilot Study" OR "Pilot Projects" OR "Pilot Project" OR "pilot" OR "implemented" OR "implementation" OR "implement*" OR "practice use" OR "practice uses" OR "clinical practice" OR "MDR application" OR "MDR applications")) **OR** ((TI("Cardiovascular Disease" OR "cerebrovascular accident" OR "Heart Infarction" OR "Brain Infarction" OR "Diabetes Mellitus" OR "cardiometabolic disease" OR "cardiometabolic diseases" OR "cardio-metabolic disease" OR "cardio-metabolic diseases" OR "cardiovascular disease" OR "cardiovascular diseases" OR "heart disease" OR "heart diseases" OR "stroke" OR "post-stroke" OR "TIA" OR "TIAs" OR "cerebrovascular accident" OR "cerebrovascular accident" OR "CBA" OR "CVAs" OR "myocardial infarction" OR "myocardial infarctions" OR "myocardial infarct*" OR "post-myocardial infarction" OR "brain infarction" OR "diabetes mellitus" OR "Diabetes" OR "IDDM" OR "NIDDM" OR "hypertension") NOT TI((exp "Infant" OR exp "Child" OR exp "Adolescent" OR "Infant" OR "Child" OR "Adolescent" OR "Infants" OR "Children" OR "Adolescents") NOT (exp "Adult" OR "adult" OR "adults" OR "elderly")) AND SU(exp "Risk" OR "risk" OR "risks" OR "atrisk" OR "outcome" OR "outcomes")) AND SU("risk stratification tools" OR "risk stratification tool" OR "population stratification tool" OR "population stratification tools" OR "population segmentation tools" OR "population segmentation tool" OR "population health management intervention" OR "population health management interventions" OR "empanelment intervention" OR "panel management intervention" OR "empanelment interventions" OR "panel management interventions" OR "risk stratification" OR "risk stratif*" OR "population stratification" OR "population stratif*" OR "population segmentation" OR "population segment*" OR exp "Population Health Management" OR "population health management" OR "population health manag*" OR "empanelment" OR "empanelment*" OR "panel management" OR "panel management*" OR "population-based approach" OR "population-based" OR "population panel" OR "population care") AND SU(exp "Pilot Study" OR "Pilot Projects" OR "Pilot Project" OR "pilot" OR "implemented" OR "implementation" OR "implement*" OR "practice use" OR "practice uses" OR "clinical practice" OR "MDR application" OR "MDR applications")) **OR** ((TI("Cardiovascular Disease" OR "cerebrovascular accident" OR "Heart Infarction" OR "Brain Infarction" OR "Diabetes Mellitus" OR "cardiometabolic disease" OR "cardiometabolic diseases" OR "cardio-metabolic disease" OR "cardio-metabolic diseases" OR "cardiovascular disease" OR "cardiovascular diseases" OR "heart disease" OR "heart diseases" OR "stroke" OR "post-stroke" OR "TIA" OR "TIAs" OR "cerebrovascular accident" OR "cerebrovascular accident" OR "CBA" OR "CVAs" OR "myocardial infarction" OR "myocardial infarctions" OR "myocardial infarct*" OR "post-myocardial infarction" OR "brain infarction" OR "diabetes mellitus" OR "Diabetes" OR "IDDM" OR "NIDDM" OR "hypertension") NOT TI((exp "Infant" OR exp "Child" OR exp "Adolescent" OR "Infant" OR "Child" OR "Adolescent" OR "Infants" OR "Children" OR "Adolescents") NOT (exp "Adult" OR "adult" OR "adults" OR "elderly")) AND SU(exp "Risk" OR "risk" OR "risks" OR "atrisk" OR "outcome" OR "outcomes")) AND TI("risk stratification tools" OR "risk stratification tool" OR "population stratification tool" OR "population stratification tools" OR "population segmentation tools" OR "population segmentation tool" OR "population health management intervention" OR "population health management interventions" OR "empanelment intervention" OR "panel management intervention" OR "empanelment interventions" OR "panel management interventions" OR (("risk stratification tools" OR "risk stratification tool" OR "population stratification tool" OR "population stratification tools" OR "population segmentation tools" OR "population segmentation tool" OR "population health management intervention" OR "population health management interventions" OR "empanelment intervention" OR "panel management intervention" OR "empanelment interventions" OR "panel management interventions") AND ("risk stratification" OR "risk stratif*" OR "population stratification" OR "population stratif*" OR "population segmentation" OR "population segment*" OR "Population Health Management" OR "population health management" OR "population health manag*" OR "empanelment" OR "empanelment*" OR "panel management" OR "panel management*")) OR (("tool" OR "tools" OR "intervention" OR "interventions") AND ("risk stratification" OR "risk stratif*" OR "population stratification" OR "population stratif*" OR "population segmentation" OR "population segment*" OR "population health management" OR "population health manag*" OR "empanelment" OR "empanelment*" OR "panel management" OR "panel management*" OR "population-based approach" OR "population-based" OR "population panel" OR "population care"))))**)** AND SU(exp "Primary Health Care" OR "Primary Health Care" OR "Primary Healthcare" OR "Primary Care" OR "Public Health" OR "Population Health" OR exp "General Practice" OR exp "General Practitioner" OR "General Practice" OR "General Practitioner" OR "General Practitioners" OR "Family Practice" OR "Family Practitioner" OR "Family Practitioners" OR "Family Physician" OR "Family Physicians" OR "Family Doctor" OR "Family Doctors" OR "Primary Care Physician" OR "Primary Care Physicians"))

KW

(**(**(KW(("Cardiovascular Disease" OR "cerebrovascular accident" OR "Heart Infarction" OR "Brain Infarction" OR "Diabetes Mellitus" OR "cardiometabolic disease" OR "cardiometabolic diseases" OR "cardio-metabolic disease" OR "cardio-metabolic diseases" OR "cardiovascular disease" OR "cardiovascular diseases" OR "heart disease" OR "heart diseases" OR "stroke" OR "post-stroke" OR "TIA" OR "TIAs" OR "cerebrovascular accident" OR "cerebrovascular accident" OR "CBA" OR "CVAs" OR "myocardial infarction" OR "myocardial infarctions" OR "myocardial infarct*" OR "post-myocardial infarction" OR "brain infarction" OR "diabetes mellitus" OR "Diabetes" OR "IDDM" OR "NIDDM" OR "hypertension") NOT ((exp "Infant" OR exp "Child" OR exp "Adolescent" OR "Infant" OR "Child" OR "Adolescent" OR "Infants" OR "Children" OR "Adolescents") NOT (exp "Adult" OR "adult" OR "adults" OR "elderly")) AND ("Risk" OR "risk" OR "risks" OR "atrisk" OR "outcome" OR "outcomes")) AND TI("risk stratification tools" OR "risk stratification tool" OR "population stratification tool" OR "population stratification tools" OR "population segmentation tools" OR "population segmentation tool" OR "population health management intervention" OR "population health management interventions" OR "empanelment intervention" OR "panel management intervention" OR "empanelment interventions" OR "panel management interventions" OR "risk stratification" OR "risk stratif*" OR "population stratification" OR "population stratif*" OR "population segmentation" OR "population segment*" OR "Population Health Management" OR "population health management" OR "population health manag*" OR "empanelment" OR "empanelment*" OR "panel management" OR "panel management*" OR "population-based approach" OR "population-based" OR "population panel" OR "population care") AND KW(exp "Pilot Study" OR "Pilot Projects" OR "Pilot Project" OR "pilot" OR "implemented" OR "implementation" OR "implement*" OR "practice use" OR "practice uses" OR "clinical practice" OR "MDR application" OR "MDR applications")) **OR** ((TI("Cardiovascular Disease" OR "cerebrovascular accident" OR "Heart Infarction" OR "Brain Infarction" OR "Diabetes Mellitus" OR "cardiometabolic disease" OR "cardiometabolic diseases" OR "cardio-metabolic disease" OR "cardio-metabolic diseases" OR "cardiovascular disease" OR "cardiovascular diseases" OR "heart disease" OR "heart diseases" OR "stroke" OR "post-stroke" OR "TIA" OR "TIAs" OR "cerebrovascular accident" OR "cerebrovascular accident" OR "CBA" OR "CVAs" OR "myocardial infarction" OR "myocardial infarctions" OR "myocardial infarct*" OR "post-myocardial infarction" OR "brain infarction" OR "diabetes mellitus" OR "Diabetes" OR "IDDM" OR "NIDDM" OR "hypertension") NOT TI((exp "Infant" OR exp "Child" OR exp "Adolescent" OR "Infant" OR "Child" OR "Adolescent" OR "Infants" OR "Children" OR "Adolescents") NOT (exp "Adult" OR "adult" OR "adults" OR "elderly")) AND KW(exp "Risk" OR "risk" OR "risks" OR "atrisk" OR "outcome" OR "outcomes")) AND KW("risk stratification tools" OR "risk stratification tool" OR "population stratification tool" OR "population stratification tools" OR "population segmentation tools" OR "population segmentation tool" OR "population health management intervention" OR "population health management interventions" OR "empanelment intervention" OR "panel management intervention" OR "empanelment interventions" OR "panel management interventions" OR "risk stratification" OR "risk stratif*" OR "population stratification" OR "population stratif*" OR "population segmentation" OR "population segment*" OR exp "Population Health Management" OR "population health management" OR "population health manag*" OR "empanelment" OR "empanelment*" OR "panel management" OR "panel management*" OR "population-based approach" OR "population-based" OR "population panel" OR "population care") AND KW(exp "Pilot Study" OR "Pilot Projects" OR "Pilot Project" OR "pilot" OR "implemented" OR "implementation" OR "implement*" OR "practice use" OR "practice uses" OR "clinical practice" OR "MDR application" OR "MDR applications")) **OR** ((TI("Cardiovascular Disease" OR "cerebrovascular accident" OR "Heart Infarction" OR "Brain Infarction" OR "Diabetes Mellitus" OR "cardiometabolic disease" OR "cardiometabolic diseases" OR "cardio-metabolic disease" OR "cardio-metabolic diseases" OR "cardiovascular disease" OR "cardiovascular diseases" OR "heart disease" OR "heart diseases" OR "stroke" OR "post-stroke" OR "TIA" OR "TIAs" OR "cerebrovascular accident" OR "cerebrovascular accident" OR "CBA" OR "CVAs" OR "myocardial infarction" OR "myocardial infarctions" OR "myocardial infarct*" OR "post-myocardial infarction" OR "brain infarction" OR "diabetes mellitus" OR "Diabetes" OR "IDDM" OR "NIDDM" OR "hypertension") NOT TI((exp "Infant" OR exp "Child" OR exp "Adolescent" OR "Infant" OR "Child" OR "Adolescent" OR "Infants" OR "Children" OR "Adolescents") NOT (exp "Adult" OR "adult" OR "adults" OR "elderly")) AND KW(exp "Risk" OR "risk" OR "risks" OR "atrisk" OR "outcome" OR "outcomes")) AND TI("risk stratification tools" OR "risk stratification tool" OR "population stratification tool" OR "population stratification tools" OR "population segmentation tools" OR "population segmentation tool" OR "population health management intervention" OR "population health management interventions" OR "empanelment intervention" OR "panel management intervention" OR "empanelment interventions" OR "panel management interventions" OR (("risk stratification tools" OR "risk stratification tool" OR "population stratification tool" OR "population stratification tools" OR "population segmentation tools" OR "population segmentation tool" OR "population health management intervention" OR "population health management interventions" OR "empanelment intervention" OR "panel management intervention" OR "empanelment interventions" OR "panel management interventions") AND ("risk stratification" OR "risk stratif*" OR "population stratification" OR "population stratif*" OR "population segmentation" OR "population segment*" OR "Population Health Management" OR "population health management" OR "population health manag*" OR "empanelment" OR "empanelment*" OR "panel management" OR "panel management*")) OR (("tool" OR "tools" OR "intervention" OR "interventions") AND ("risk stratification" OR "risk stratif*" OR "population stratification" OR "population stratif*" OR "population segmentation" OR "population segment*" OR "population health management" OR "population health manag*" OR "empanelment" OR "empanelment*" OR "panel management" OR "panel management*" OR "population-based approach" OR "population-based" OR "population panel" OR "population care"))))**)** AND KW(exp "Primary Health Care" OR "Primary Health Care" OR "Primary Healthcare" OR "Primary Care" OR "Public Health" OR "Population Health" OR exp "General Practice" OR exp "General Practitioner" OR "General Practice" OR "General Practitioner" OR "General Practitioners" OR "Family Practice" OR "Family Practitioner" OR "Family Practitioners" OR "Family Physician" OR "Family Physicians" OR "Family Doctor" OR "Family Doctors" OR "Primary Care Physician" OR "Primary Care Physicians"))

AB

(**(**(AB(("Cardiovascular Disease" OR "cerebrovascular accident" OR "Heart Infarction" OR "Brain Infarction" OR "Diabetes Mellitus" OR "cardiometabolic disease" OR "cardiometabolic diseases" OR "cardio-metabolic disease" OR "cardio-metabolic diseases" OR "cardiovascular disease" OR "cardiovascular diseases" OR "heart disease" OR "heart diseases" OR "stroke" OR "post-stroke" OR "TIA" OR "TIAs" OR "cerebrovascular accident" OR "cerebrovascular accident" OR "CBA" OR "CVAs" OR "myocardial infarction" OR "myocardial infarctions" OR "myocardial infarct*" OR "post-myocardial infarction" OR "brain infarction" OR "diabetes mellitus" OR "Diabetes" OR "IDDM" OR "NIDDM" OR "hypertension") NOT ((exp "Infant" OR exp "Child" OR exp "Adolescent" OR "Infant" OR "Child" OR "Adolescent" OR "Infants" OR "Children" OR "Adolescents") NOT (exp "Adult" OR "adult" OR "adults" OR "elderly")) AND ("Risk" OR "risk" OR "risks" OR "atrisk" OR "outcome" OR "outcomes")) AND TI("risk stratification tools" OR "risk stratification tool" OR "population stratification tool" OR "population stratification tools" OR "population segmentation tools" OR "population segmentation tool" OR "population health management intervention" OR "population health management interventions" OR "empanelment intervention" OR "panel management intervention" OR "empanelment interventions" OR "panel management interventions" OR "risk stratification" OR "risk stratif*" OR "population stratification" OR "population stratif*" OR "population segmentation" OR "population segment*" OR "Population Health Management" OR "population health management" OR "population health manag*" OR "empanelment" OR "empanelment*" OR "panel management" OR "panel management*" OR "population-based approach" OR "population-based" OR "population panel" OR "population care") AND AB(exp "Pilot Study" OR "Pilot Projects" OR "Pilot Project" OR "pilot" OR "implemented" OR "implementation" OR "implement*" OR "practice use" OR "practice uses" OR "clinical practice" OR "MDR application" OR "MDR applications")) **OR** ((TI("Cardiovascular Disease" OR "cerebrovascular accident" OR "Heart Infarction" OR "Brain Infarction" OR "Diabetes Mellitus" OR "cardiometabolic disease" OR "cardiometabolic diseases" OR "cardio-metabolic disease" OR "cardio-metabolic diseases" OR "cardiovascular disease" OR "cardiovascular diseases" OR "heart disease" OR "heart diseases" OR "stroke" OR "post-stroke" OR "TIA" OR "TIAs" OR "cerebrovascular accident" OR "cerebrovascular accident" OR "CBA" OR "CVAs" OR "myocardial infarction" OR "myocardial infarctions" OR "myocardial infarct*" OR "post-myocardial infarction" OR "brain infarction" OR "diabetes mellitus" OR "Diabetes" OR "IDDM" OR "NIDDM" OR "hypertension") NOT TI((exp "Infant" OR exp "Child" OR exp "Adolescent" OR "Infant" OR "Child" OR "Adolescent" OR "Infants" OR "Children" OR "Adolescents") NOT (exp "Adult" OR "adult" OR "adults" OR "elderly")) AND AB(exp "Risk" OR "risk" OR "risks" OR "atrisk" OR "outcome" OR "outcomes")) AND AB("risk stratification tools" OR "risk stratification tool" OR "population stratification tool" OR "population stratification tools" OR "population segmentation tools" OR "population segmentation tool" OR "population health management intervention" OR "population health management interventions" OR "empanelment intervention" OR "panel management intervention" OR "empanelment interventions" OR "panel management interventions" OR "risk stratification" OR "risk stratif*" OR "population stratification" OR "population stratif*" OR "population segmentation" OR "population segment*" OR exp "Population Health Management" OR "population health management" OR "population health manag*" OR "empanelment" OR "empanelment*" OR "panel management" OR "panel management*" OR "population-based approach" OR "population-based" OR "population panel" OR "population care") AND AB(exp "Pilot Study" OR "Pilot Projects" OR "Pilot Project" OR "pilot" OR "implemented" OR "implementation" OR "implement*" OR "practice use" OR "practice uses" OR "clinical practice" OR "MDR application" OR "MDR applications")) **OR** ((TI("Cardiovascular Disease" OR "cerebrovascular accident" OR "Heart Infarction" OR "Brain Infarction" OR "Diabetes Mellitus" OR "cardiometabolic disease" OR "cardiometabolic diseases" OR "cardio-metabolic disease" OR "cardio-metabolic diseases" OR "cardiovascular disease" OR "cardiovascular diseases" OR "heart disease" OR "heart diseases" OR "stroke" OR "post-stroke" OR "TIA" OR "TIAs" OR "cerebrovascular accident" OR "cerebrovascular accident" OR "CBA" OR "CVAs" OR "myocardial infarction" OR "myocardial infarctions" OR "myocardial infarct*" OR "post-myocardial infarction" OR "brain infarction" OR "diabetes mellitus" OR "Diabetes" OR "IDDM" OR "NIDDM" OR "hypertension") NOT TI((exp "Infant" OR exp "Child" OR exp "Adolescent" OR "Infant" OR "Child" OR "Adolescent" OR "Infants" OR "Children" OR "Adolescents") NOT (exp "Adult" OR "adult" OR "adults" OR "elderly")) AND AB(exp "Risk" OR "risk" OR "risks" OR "atrisk" OR "outcome" OR "outcomes")) AND TI("risk stratification tools" OR "risk stratification tool" OR "population stratification tool" OR "population stratification tools" OR "population segmentation tools" OR "population segmentation tool" OR "population health management intervention" OR "population health management interventions" OR "empanelment intervention" OR "panel management intervention" OR "empanelment interventions" OR "panel management interventions" OR (("risk stratification tools" OR "risk stratification tool" OR "population stratification tool" OR "population stratification tools" OR "population segmentation tools" OR "population segmentation tool" OR "population health management intervention" OR "population health management interventions" OR "empanelment intervention" OR "panel management intervention" OR "empanelment interventions" OR "panel management interventions") AND ("risk stratification" OR "risk stratif*" OR "population stratification" OR "population stratif*" OR "population segmentation" OR "population segment*" OR "Population Health Management" OR "population health management" OR "population health manag*" OR "empanelment" OR "empanelment*" OR "panel management" OR "panel management*")) OR (("tool" OR "tools" OR "intervention" OR "interventions") AND ("risk stratification" OR "risk stratif*" OR "population stratification" OR "population stratif*" OR "population segmentation" OR "population segment*" OR "population health management" OR "population health manag*" OR "empanelment" OR "empanelment*" OR "panel management" OR "panel management*" OR "population-based approach" OR "population-based" OR "population panel" OR "population care"))))**)** AND AB(exp "Primary Health Care" OR "Primary Health Care" OR "Primary Healthcare" OR "Primary Care" OR "Public Health" OR "Population Health" OR exp "General Practice" OR exp "General Practitioner" OR "General Practice" OR "General Practitioner" OR "General Practitioners" OR "Family Practice" OR "Family Practitioner" OR "Family Practitioners" OR "Family Physician" OR "Family Physicians" OR "Family Doctor" OR "Family Doctors" OR "Primary Care Physician" OR "Primary Care Physicians"))

# Supplementary data

**RE-AIM Data Extraction Components and Descriptions**

*adapted from Racey, M., Markle-Reid, M., Fitzpatrick-Lewis, D. et al. Applying the RE-AIM implementation framework to evaluate fall prevention interventions in community dwelling adults with cognitive impairment: a review and secondary analysis. BMC Geriatr 21, 441 (2021). https://doi.org/10.1186/s12877-021-02376-7

| **Author, Year, Country** | Self explanatory |
| --- | --- |
| **Target Population** | Brief description of the targeted population |
| **Study location** | The location(s) where the intervention is delivered. |
| **Purpose** | What is the purpose of the manuscript; provide details that will give us highlights of the paper (e.g., the purpose of this paper was to report on the short term effectiveness of a dietary program, with special attention on the feasibility (adoption) of the program in a clinical setting). |
| **Study Design** | Randomized controlled trial (RCT), clinical controlled trial (CCT), Observational, etc. |
| **Year(s) data collection** | Years of data collection |

| **Reach** | The proportion & representativeness of individuals willing to participate in a given intervention |
| --- | --- |
| **Described target population** | A brief description of the broader target population (i.e., not simply of the study sample). |
| **Method to identify target population** | Explain the method employed to ascertain the target population suitable for inclusion in the research endeavor. |
| **Recruitment Strategies** | Describe the methods used to recruit participants into the study. |
| **Inclusion criteria** | Provide a clear delineation of the attributes of the target population that were utilised to ascertain the eligibility of prospective participants for study inclusion |
| **Exclusion criteria** | Offer a precise articulation of the specific traits that would render a potential participant ineligible for study participation, accompanied by the inclusion of the percentage of individuals excluded based on these characteristics. |
| **Participation rate** | Sample size divided by the target population denominator.  Example: 200 (number of people agree to participate)/250 (number of eligible participants contacted for participation) = 80% |
| **Cost of recruitment** | The cost of recruitment can reflect monetary and/or time units. |
| **Use of qualitative methods to measure reach** | Presenting non-quantitative dimensions of reach through the utilization of verbal expressions, sentences, descriptive accounts, or codes. Common methodologies encompass key informant interviews, focus groups, and field notes. |

| **Efficacy/Effectiveness** | The influence of an intervention on important outcomes, including potential negative effects, quality of life, & economic outcomes |
| --- | --- |
| **Quality of life measure** | Incorporates an assessment of the quality of life, allowing for a certain degree of flexibility in categorizing publications that address well-being or contentment with life. |
| **Measure unintended consequences (negative) and results** | To assess unforeseen outcomes and consequences that may arise from the intervention and potentially lead to unintended adverse effects. |
| **Cost effectiveness** | Code as reported if specific mention and amounts are provided for the cost of the intervention. |
| **Use of qualitative methods to measure efficacy/effectiveness** | Gathering qualitative input from participants regarding the extent to which they perceived the intervention as effective or successful. Common methodologies employed for this purpose include focus groups, interviews, and the use of diaries (in the form of textual entries or visual representations) |

| **Adoption – Diffusion – Setting Level** | The proportion & representativeness of locations willing to initiate & adopt an intervention |
| --- | --- |
| **Participation rate** | The proportion of sites eligible and contacted that participated. |
| **Description of targeted location** | Characteristics that would be considered an ideal location for the intervention. |
| **Inclusion/exclusion criteria of setting** | The explicit statement of characteristics of the setting that were used to determine if a potential setting is eligible to participate. |
| **Description of intervention location** | The explicit statement of characteristics of the location of the intervention. |
| **Method to identify setting** | Describe the process by which the location was identified for participation in the study. |
| **Average Number of persons served per setting** | Calculated average number of participants at each site. |

| **Adoption – Diffusion – Staff Level** | The proportion & representativeness intervention staff willing to initiate & adopt an intervention |
| --- | --- |
| **Participation rate** | The proportion of the staff that was eligible and contacted and participated. |
| **Method to identify target delivery agent** | Elucidate the procedure employed to determine the selection of the target delivery agent for inclusion in the study. |
| **Level of expertise of delivery agent** | The inclusion criteria for delivery agents in terms of their training or educational background in the relevant field, including degrees, certifications (e.g., PhD, Masters, Registred Nurse etc.). |
| **Inclusion/exclusion criteria of delivery agent** | Provide a clear articulation of the specific attributes of the delivery agents that were utilised to ascertain the eligibility of prospective agents for participation in the study. |
| **Use of qualitative methods to measure adoption** | Used qualitative methods to understand the process of adoption. |

| **Implementation** | How consistently various elements of an intervention are delivered as intended by intervention staff, & the time & cost of the intervention |
| --- | --- |
| **Theories** | Present a precise declaration of the theories or principles employed in the development of the intervention, such as social cognitive theory, theory of planned behavior, and others. |
| **Engagement** | Engagement of patient, caregiver, or otherwise, to develop or inform the intervention, post-intervention, etc |
| **Intervention number of contacts** | Total number of encounters with participants for the intervention arm. Could include face-to-face meetings, telephone calls, newsletters etc. |
| **Timing of contacts** | Describe when the intervention contacts occur over the course of the intervention. |
| **Duration of contacts** | Length of each intervention contact. |
| **Consistency of implementation across setting and delivery agents** | Description of the degree of similarities between multiple settings sites & delivery agents |
| **Measure of cost** | The ongoing cost of delivery across all levels of the intervention. |
| **Use of qualitative methods to measure implementation** | Used qualitative methods to understand the process of implementation.  Example: focus groups, interviews |

| **Maintenance** | The extent to which participants make & maintain a behavior change & the sustainability of a program or policy in the setting in which it was intervened |
| --- | --- |
| **Was individual behaviour assessed at some duration following the completion of the intervention? (give the duration of follow-up)** | Provide a detailed account of the outcome measures used to assess individuals at a specific duration after the termination of the intervention, thereby capturing the follow-up data. |
| **Attrition** | Detail the extent to which participants were no longer available for follow-up assessment, including the reasons for their attrition, during the period spanning from the completion of the intervention to the follow-up stage. |
| **Use of qualitative methods to measure individual maintenance** | Used qualitative methods to understand the process of individual level maintenance (during follow-up period) of changes to the primary outcome.  Example: focus groups, interviews |
| **Is the program still in place?** | Description of program continuation after completion of the research study. |
| **If no: reason for discontinuation** | Description of why the intervention was terminated |
| **If yes: was the program modified? Specify** | Description of any changes that were made to the original program |
| **Attrition** | Elucidate the extent to which sites were unable to be tracked for follow-up, along with the reasons for their attrition, during the period from the completion of the intervention to the follow-up stage. |
| **Use of qualitative methods to measure organisational level maintenance** | Used qualitative methods to understand the process of intervention sustainability at the organizational level |
